# Soft fibers with magnetoelasticity for wearable electronics

**DOI:** 10.1038/s41467-021-27066-1

**Published:** 2021-11-19

**Authors:** Xun Zhao, Yihao Zhou, Jing Xu, Guorui Chen, Yunsheng Fang, Trinny Tat, Xiao Xiao, Yang Song, Song Li, Jun Chen

**Affiliations:** Department of Bioengineering, University of California, Los Angeles, Los Angeles, CA 90095 USA

**Keywords:** Biomedical engineering, Polymers, Magnetic properties and materials

## Abstract

Magnetoelastic effect characterizes the change of materials’ magnetic properties under mechanical deformation, which is conventionally observed in some rigid metals or metal alloys. Here we show magnetoelastic effect can also exist in 1D soft fibers with stronger magnetomechanical coupling than that in traditional rigid counterparts. This effect is explained by a wavy chain model based on the magnetic dipole-dipole interaction and demagnetizing factor. To facilitate practical applications, we further invented a textile magnetoelastic generator (MEG), weaving the 1D soft fibers with conductive yarns to couple the observed magnetoelastic effect with magnetic induction, which paves a new way for biomechanical-to-electrical energy conversion with short-circuit current density of 0.63 mA cm^−2^, internal impedance of 180 Ω, and intrinsic waterproofness. Textile MEG was demonstrated to convert the arterial pulse into electrical signals with a low detection limit of 0.05 kPa,  even with heavy perspiration or in underwater situations without encapsulations.

## Abstract (web summary)

The authors invented a textile magnetoelastic generator, weaving 1D soft fibers with conductive yarns to couple the observed magnetoelastic effect with magnetic induction, which paves a new way for biomechanical-to-electrical energy conversion.

## Introduction

Magnetoelastic effect is found in rigid and 3D metal alloys such as Fe_1−*x*_Co_*x*_^1^, Tb_*x*_Dy_1−*x*_Fe_2_ (Terfenol-D)^2^, and Ga_*x*_Fe_1−*x*_ (Galfenol)^3^, which are usually used for civil engineering for building vibration control with an applied magnetic field^4^. This effect has been restrained from the field of soft-matter electronics for three reasons: (1) the rigid metal alloys hold six orders higher mechanical modulus than human bodies, (2) the required mechanical stress for the magnetoelastic alloys is beyond the range of biomechanical stress, and (3) they rely on external magnetic fields, resulting in a bulky structure.

Textiles^5,6^, one of the earliest human inventions, have become an indispensable part of our lives owing to their unique properties, such as light weight, touching softness, and inherent breathability^7–12^. Merging textiles and electronics is a compelling approach to realize smart textiles with additional values while maintaining wearing comfort^10–12^. Biomechanical motions provide clean and renewable energy sources^13^. Fiber-based textiles can effectively accommodate the body-motion-induced complex deformation for electricity generation, which is an essential pathway to build up human-centered self-powered bioelectronics. For example, the current widely-adopted biomechanical energy harvesting textiles, based on triboelectric effect^13–19^ and piezoelectric effect^20–24^, display promising performance in energy, sensing, and therapeutics^25,26^. For practical on-body applications, the ability to withstand ambient humidity caused by perspiration and fluidic environment of the human body is an essential property for wearable textile-based devices. An encapsulation layer would enhance the devices’ humidity resistance. However, it usually compromises their electric output performance, undermining textile breathability and wearability^27^.

Herein, we observed the magnetoelastic effect arising from magnetic dipole alignment in soft magnetic fibers, which demonstrated stronger magnetomechanical coupling than that observed in the conventional rigid metal alloys. Previously, magnetorheological elastomers^28,29^ have been reported for vibration absorbers^30,31^, actuators^32,33^, and soft robots^34,35^, based on their magnetostriction property and tunable mechanical properties. Most attention has been focused on the magnetostriction-enabled shapes or dimensions changing during the process of magnetic actuation^30,36^ and adjusting stiffness and shear modulus under an applied magnetic field^37^ as detailly explained in Supplementary Note 1. Not until very recently, the giant magnetoelastic effect was observed in a thin soft composite membrane^38^. In this article, via manipulating the magnetic dipole–dipole interaction under mechanical deformation, we also observed the magnetoelastic effect in one-dimensional (1D) soft fibers with a collection of compelling advantages: (1) magnetoelastic effect is realized in soft fibers with Young’s modulus around 630 kPa, which is mechanically compatible with human body, (2) the applied pressure that needs to deform the 1D soft fiber is within the range of 450 kPa and obtainable by human daily activities, and (3) it requires no external magnetic field. These features endow the 1D soft fibers wide-range of applicability in building up soft-matter technologies. To demonstrate, a textile magnetoelastic generator (MEG) was developed, relying on a two-step conversion mechanism that couples the magnetoelastic effect and magnetic induction. A short-circuit current density of 0.63 mA cm^−2^ and an internal impedance of 180 Ω were achieved. Since magnetic fields can penetrate water with negligible intensity loss, the textile MEG is intrinsically humidity-resistant without additional encapsulation. The wearable textile MEG was also applied to convert the arterial pulse into electrical signals, under the circumstance of heavy body perspiration, for the measurements of self-powered cardiovascular parameters. A customized cellphone application (APP) based on a built-in algorithm, was also developed for one-click health data sharing and data-driven diagnosis. With features like intrinsic humidity-resistance, high current, and low internal resistance, we foresee that the magnetoelastic effect in 1D fibers could be used as the building blocks for soft-matter electronics with a wide-range of applications in energy, medical, robotics, and artificial perception fields.

## Results

### Design of soft magnetic fibers

**Fig. 1:**
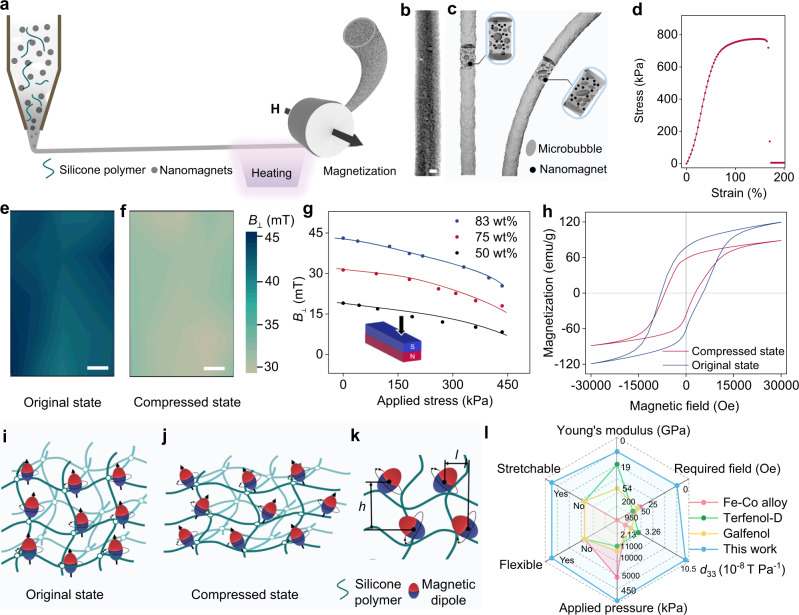
The magnetoelastic effect in soft fibers. **a** Schematic of the fabrication process of the soft magnetic fibers. **b** Micro-CT image and **c** Schematics of the soft magnetic fiber. Scale bar: 0.4 mm. **d** Stress–strain curves of the soft magnetic fibers with 83 wt% magnetic concentration. **e**, **f** Vertical magnetic flux density (Δ*B*_⊥_) mapping of the soft magnetic fiber on south pole surface in the original state (**e**) and under a compressed pressure of 300 kPa (**f**). Scale bars: 0.24 mm. **g** Magnetic field variation of the soft magnetic fiber under applied stress on the south pole surface. **h** Magnetic hysteresis loop of the soft magnetic fiber in original state and in compressed state. **i**, **j** Schematics of the magnetic dipole distribution in soft magnetic fiber of initial state (**i**) and compressed state (**j**). **k** Schematic of the wavy chain model. **l** Performance comparison of the soft magnetic fibers with rigid metal alloys.

The fabrication procedure of the 1D soft fibers with magnetoelastic effect was schematically illustrated in Fig. 1a. Solid nanomagnets were dispersed in the highly viscous liquid silicone polymer. Air microbubbles were systematically introduced into the composite during mechanical stirring to form a three-phase mixture, which will further go through an extrusion processing via an adjustable nozzle. The soft magnetic fibers with controllable diameters were obtained by crosslinking the three-phase mixture during the heating process. As demonstrated in the cross-section view of the scanning electron microscope (SEM) image and the histogram of the inter-particle distance, magnetic particles were dispersed in the silicone polymer with a mean distance of 14.69 μm and a standard deviation of 4.09 μm (Supplementary Fig. 1). Microbubbles co-exist and play a critical role in bringing porosities into the soft magnetic fibers. On the one hand, the outmost microbubbles were broken to form surface roughness on the fibers, improving the softness, touching comfort, and stretchability. On the other hand, it helps reduce the overall fiber density. Micro-computed tomography (Micro-CT) was employed to characterize internal structure of the fiber. As shown in Fig. 1b, there are uniformly distributed microbubbles-induced nanoscale-to-microscale cavities in the soft magnetic fibers, which are consistent with all the three cross-section views shown in Supplementary Fig. 2. This observation is also justified by the overall morphology of the magnetic fibers showing in a dynamic 3D model created by the Micro-CT (Supplementary Movie 1). Owing to the designed microbubble cavities, the soft magnetic fibers could be effectively deformed under gentle bending and compressing (Fig. 1c). As shown in Supplementary Fig. 3a–c, by controlling the magnetic concentrations, the magnetic fibers show different mechanical properties. Hence, the 83 wt% soft magnetic fiber with a stretchability up to 180% and a Young’s modulus of 630 kPa was chosen (Fig. 1d), for it holds similar mechanical properties to human tissues^39^ and can ensure its adaptability to human skin^39,40^. Apart from the advantages in mechanical properties, the 83 wt% soft magnetic fibers have larger coercive field of 7.89 kOe and remnant magnetization of 76 emu g^−1^ than those of the magnetic fibers with 75 and 50 wt% magnetic concentrations (Supplementary Fig. 3d). Moreover, it is worth noting that the soft magnetic fibers could also be massively produced. Three spools of fibers could be obtained via one-time machine extrusion (Supplementary Fig. 4).

To reorient the magnetic dipoles in the fibers, an adjustable impulse field, *H*, was employed for magnetization. The magnetic dipoles were aligned in the soft polymer matrix with uniform polarity. In order to investigate the magnetic field variation under mechanical deformation, a two-axial motion platform was established to visualize the magnetic field variation under different applied stress, as schematically illustrated in Supplementary Fig. 5. And the magnetic flux density on the south pole, north pole, and side surface of the soft fibers were systematically investigated. Figure 1e shows the magnetic flux density mapping on the south pole of the soft fiber in its initial state, and Fig. 1f shows the mapping image in its compressed state under a pressure of 300 kPa. A comparative study indicates that the magnetic flux density decreased in both the edge and the middle of the fiber under applied stress. A similar trend was also observed for the magnetic flux density mapping on the north pole of the 1D fiber (Supplementary Fig. 6). We also investigated the values of magnetic field variation with different nanomagnet concentrations. As shown in Fig. 1g, under a constant stress, soft magnetic fibers with 83 wt% nanomagnet concentration have the largest values of magnetic field variation of 18 mT than those of 75 wt% (13 mT) and 50 wt% (11 mT) nanomagnet concentrations. As a result, a maximum magnetomechanical coupling factor of 8.28 × 10^−8^ T Pa^−1^ was derived from the magnetic field variation of the 1D fibers, corresponding to a 6.6-time enhancement compared to that reported in the traditional bulky metal alloys. As for the side surface, the magnetic flux density mapping images are shown in Supplementary Fig. 7, and the magnetic field variation (Δ*B*_⊥_) data are plotted in Supplementary Fig. 8a. The Δ*B*_⊥_ are larger in the edge than that in the middle of the magnetic fiber under a fixed applied pressure. And a maximum magnetomechanical coupling factor of 1.05 × 10^−7^ T Pa^−1^ was derived from the edge of the side surface, corresponding to an 8.4-time enhancement compared to that reported in the traditional bulky metal alloys (Supplementary Fig. 8b). Furthermore, magnetic hysteresis loop of the soft magnetic fiber in original state and compressed state are tested. As shown in Fig. 1h, both the remnant magnetization and coercive field of the magnetic fiber decreased in compressed state, which may be due to the rearrangement of the nanomagnets inside the soft magnetic fibers. This result is consistent with the decreasing magnetic flux density on the surface of the magnetic fibers.

More importantly, we verified the magnetoelastic effect in different magnetic materials and found that the magnetoelastic effect arising from magnetic dipole alignment in soft systems is a universal phenomenon. Soft magnetic fibers made of SrFe_12_O_19_ and Fe_3_O_4_ also demonstrated magnetic field decrease under mechanical deformation, which is similar to that made by NdFeB nanomagnets (Supplementary Fig. 8c). In order to compare their mechanical and magnetic properties, we have summarized their magnetomechanical coupling factor and Young’s modulus in Supplementary Table 1.

### Wavy chain analytical model

To explain the mechanical-induced magnetic field variation mechanism and ultra-strong magnetomechanical coupling in the soft magnetic fibers, a wavy chain analytical model was established based on the magnetic dipole–dipole interaction and demagnetizing factor. As displayed in Fig. 1i and j, the nanomagnets are considered to be single magnetic dipoles and aligned in a wavy chain structure in the initial state after impulsed magnetization. The vertical and horizontal distance between neighboring magnetic particles inside the wavy chain structure are denoted as *h* and *l*, respectively (Fig. 1k). In the compressed state, the nanomagnet chain structure varies and alters the dipole–dipole interaction inside the chain as well as the demagnetizing field of the chain structure. The coupling and mutual interaction of these two factors contribute to the decrease of surface magnetic flux density. Once the uniaxial stress is released, the recovery of the nanomagnet wavy chain structure reverses the magnetic flux density back. Quantitively, the magnetic field variation in response to applied mechanical pressure can be evaluated by *H*_1⊥_/*H*_0⊥_ (equals to *B*_1⊥_/*B*_0⊥_), which can be expressed as a function of principle stretch *λ* based on the wavy chain model (Supplementary Note 2) as below

1 $${H}_{1\perp }/{H}_{0\perp }\approx \frac{\frac{1}{\chi}-\frac{k}{2a{\lambda }^{1.5}+1}+\frac{{r}^{3}}{3{\lambda }^{3}{h}^{3}}\left(0.3006-f\left(\frac{l}{h{\lambda }^{1.5}}\right)\right)}{\frac{1}{\chi}-\frac{k}{\left(2a+1\right)}+\frac{{r}^{3}}{3{h}^{3}}\left(0.3006-f\left(\frac{l}{h}\right)\right)}$$

where *H*_1⊥_ and *H*_0⊥_ represent the vertical magnetic field with and without applied mechanical pressure, respectively, and *r* is the radius of the nanomagnet. χ represents the contribution of remnant magnetization. *a* is the estimated aspect ratio of a single wavy chain structure. *h* and *l* denote the vertical and horizontal distances between two adjacent magnetic dipoles in the wavy chain, respectively. 0.3006*−f*(*x*) is the dipole alignment factor describing the contribution of all other magnetic dipoles to a single dipole in the wavy chain on the surface of the fiber. *k* represents a constant, characterizing the influence of nonideality, neighboring chain–chain interaction, and macroscopic shape effect to the demagnetizing factor under compressive deformation. The derivation of the dipole alignment factor is detailed in Supplementary Note 2. It should be noted that Eq. (1) differs slightly from our previous work by including the contribution of remnant magnetization and applying the strain effect on aspect ratio *a* instead of demagnetizing factor $\frac{1}{2a+1}$, which is physically more reasonable and would yield more accurate results when *a* is small^38^. The magnetic field variation *H*_1⊥_/*H*_0⊥_ is further linked to the compressive stress *s* through an incompressible Neo-Hookean material model with the following equation:

2 $$s=G(\lambda -1/{\lambda }^{2})$$

where *G* is the shear modulus of the soft magnetic fiber. With an estimated value of χ = 14.99, *a* = 105, *r* = 2.5 µm, *h* = 13.5 µm and *l* = 14.85 µm in the soft magnetic fiber. We found that when *k* = 3.8, the wavy chain model accurately captures its magnetic field variation in response to the compressive stress changing from 0 to 450 kPa, which is well consistent with the experimental observation (Supplementary Fig. 9). With both experimental results and theoretical verification, it is clear that the compressive stress on the order of 100 kPa was enough to generate a significant magnetic field variation up to ~18 mT in the 1D soft magnetic fiber.

Besides the magnetomechanical coupling analysis, we took a further step to comprehensively compare the 1D soft fibers with Fe–Co alloys, Terfenol-D, and Galfenol in other parameters, which were reported to show the strongest magnetoelastic effect, as summarized by the results in Fig. 1l. Towards building up soft matter technologies, the 1D soft magnetic fibers show great advantages over the two metal alloys in all the six performance indexes, including magnetomechanical coupling factor *d*_33_ (T/Pa), Young’s modulus (kPa), flexibility, stretchability, applied stress (kPa), and required magnetic field (A/m). Specifically, (1) the maximum magnetomechanical coupling factor (*d*_33_) of soft fibers shows up to 8.4-time enhancement than that in the metal alloys, (2) the soft fibers are flexible and stretchable with Young’s modulus of 630 kPa, while metal alloys are bulky and rigid with Young’s modulus of up to 200 GPa, (3) the applied pressure on the soft fibers is below 450 kPa, which is much lower than the typical value (5–11 MPa) of conventional metal alloys, and (4) the soft fiber requires no external magnetic field to perform mechanical-to-magnetic conversation, while metal alloy demands external magnetic field up to 950 Oe via external permanent magnets or bulky electromagnets. With a collection of these compelling features, the developed 1D soft fibers with magnetoelastic effect show great potential in mechanical-to-magnetic conversion in a soft manner. And it could be further coupled with other effects, including but not limited to magnetic induction, magneto-optic effect, and magnetocaloric effect for a wide range of applications in electricity generation, optical regulation, and thermal management.

### Constructing a textile MEG

**Fig. 2:**
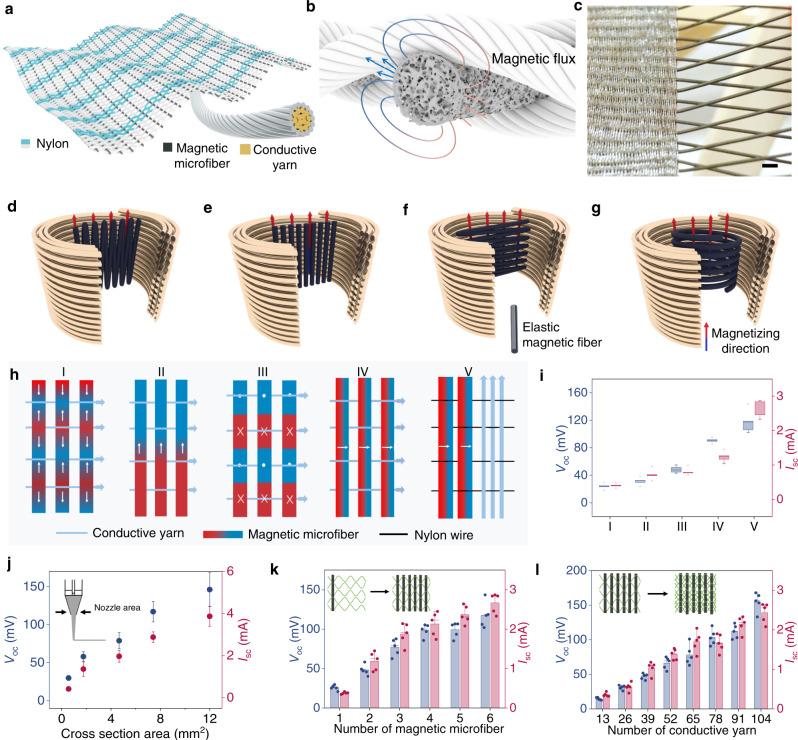
Designing a textile MEG. **a** Schematic design of the textile MEG. **b** Schematics of the working mechanism of the textile MEG. **c** Photograph showing the weaving processing with a loom. Scale bar: 1 cm. **d**–**g** Schematics of parallel folded magnetic fiber (**d**), straight magnetic fiber (**e**), perpendicular folded magnetic fiber (**f**), and annular folded magnetic fiber (**g**) with an applied magnetic field. **h** Schematics of textile MEGs with five folding patterns. **i** Dependence of the short-circuit current (*I*_sc_) and open-circuit voltage (*V*_oc_) of the textile MEGs on different folding patterns. The boxplots show maximum, minima, median and upper and lower quartiles. **j**–**l** Dependence of the electrical output of the textile MEGs on (**j**) the cross-section area of the magnetic fibers. Data are mean ± SD; *n* = 5. **k** The number of the magnetic fibers. Each dot represents the result of a single test. Data are mean ± SD; *n* = 5. **l** The number of the conductive yarns. Each dot represents the result of a single test. Data are mean ± SD; *n* = 5.

To demonstrate the viability of the observed magnetoelastic effect in 1D soft fibers, we further coupled it with the magnetic induction to develop a wearable textile MEG as soft-matter electronics for on body electricity generation. The textile MEG is formed by interlacing the magnetic fibers with the conductive yarns, as illustrated in Fig. 2a. The conductive yarns were consisted of silver-coated nylon fibers and nylon fibers winded by a braiding machine (Supplementary Fig. 10a), and they are amenable to large-scale fabrication (Supplementary Fig. 10b). The silver coated on the nylon microfiber is very uniform to assure the fiber conductivity, as the SEM image demonstrated in Supplementary Fig. 11. The electricity generation of the textile MEG in response to applied force relies on a two-step energy conversion, namely, mechanical-to-magnetic and magnetic-to-electrical conversions. The deformation of soft magnetic fibers was used to efficiently create magnetic fields variation under mechanical stimulus, which was then converted into electricity by using the conductive yarns based on magnetic induction. Structurally, the conductive yarns are woven with porous magnetic fibers within the textile MEG. The two-step conversion happens at each yarn intersection when the textile is clapped, waved, or folded, as schematically illustrated in Fig. 2b. It is worth noting that the textile MEG could be massively produced by a weaving loom (Fig. 2c).

### Parameter optimization

To achieve the optimized mechanical-to-electrical energy conversion, a number of designing parameters were systematically investigated in the textile MEG. To begin with, in the first step of mechanical-to-magnetic conversion, the magnetization pattern of the soft magnetic fibers plays an important role in creating efficient magnetic field variation. Thus, magnetic fibers with different magnetic domain distribution were compared. In order to program the magnetic fibers with controllable magnetic domain, the fiber was bent to different shapes under a constant magnetizing field (Fig. 2d–g). The corresponding magnetic domain distributions in the soft fibers are demonstrated in Fig. 2h, in which the folding patterns of mode I–III were programmed with magnetization direction depicted in Fig. 2d–f, respectively, and the folding patterns of modes IV and V were programmed using magnetization direction depicted in Fig. 2g. Their electric outputs were systematically investigated as shown in Fig. 2i. From mode I to IV, when the magnetic fibers were vertically interlacing with the conductive yarns, the magnetic domain distribution in mode IV showed the highest electrical output, owing to the uniform domain distribution in the magnetic fibers. However, when two kinds of yarns were interlacing in parallel with each other as shown in mode V, it demonstrated even better electrical performance than mode IV, because it can accommodate more magnetic field variation. Additionally, the cross-sectional area and number of the magnetic fibers also have great influence on the magnetic field variation in the textile. As illustrated in Fig. 2j, the textile MEG has better electrical output with larger cross-section area of magnetic fibers, since larger magnetic flux variation can be created in thicker magnetic fibers under mechanical excitation. Besides the fiber diameter, the electrical output of the textile MEG could also be largely boosted with additional fiber numbers. As shown in Fig. 2k, both the current and voltage increase linearly with the number of woven magnetic fibers.

Furthermore, the number of the conductive yarns was also investigated because it is critical to the second step of magnetic-to-electrical conversion. As demonstrated in Fig. 2l, the electrical output increases linearly with an increase in the number of conductive yarns. This corresponds to Faraday’s law since the number of conductive yarns is directly proportional to the induced voltage. As a result, the electrical output of the textile MEG is designable with the folding patterns, and the number of both magnetic fibers and conductive yarns.

### Textile MEG for energy

Harvesting energy from biomechanical motions with a textile provides a pervasive and convenient energy solution for body-centered electronics while maintaining wearing comfort. Humidity-resistance is an indispensable feature of on-body applications since sweat glands on human skins can perspire up to 3.5 L h^−1^ in a human’s daily exercise^41^. Adding an additional encapsulation layer could achieve certain humidity-resistance at the price of undermining the electric output and wearability^27^. On the basis of magnetic dipole manipulation, the fundamentally new textile MEG is intrinsically waterproof since the magnetic fields are able to penetrate water with negligible intensity loss.

**Fig. 3:**
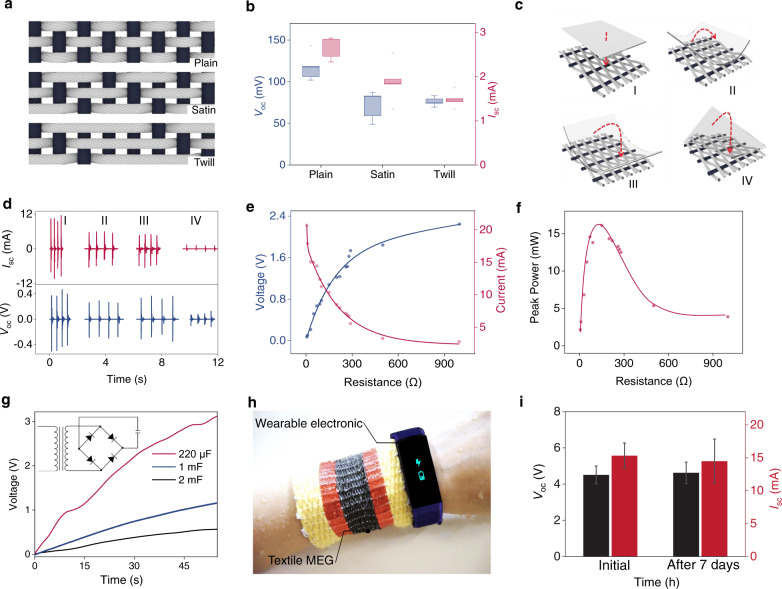
Intrinsically waterproof textile for wearable power generation. **a**, **b** Schematics showing the three different weaving patterns (**a**) and the corresponding measured electrical signals (**b**) of the textile MEG. Boxplots show maximum, minima, median, and upper and lower quartiles. **c**, **d** Schematics showing four excitation modes of the textile MEG (**c**) and the corresponding measured electrical signals (**d**). **e** Dependence of the output voltage and current of the textile MEG on the external load resistances. **f** Dependence of the power on the external load resistances. **g** Charging commercial capacitors up to 3 V. **h** Schematic of the textile MEG mixed with wool fibers driving a wearable biosensor system. **i** Electric outputs of the textile MEG before and after being submerged in water for 7 days. Data are mean ± SD; *n* = 5.

Towards practical application, the weaving patterns of the textile were systematically investigated. This is a necessary designing parameter for consideration since it shows impact on both the electrical output and the appearance of the textile MEG. As illustrated in Fig. 3a and Supplementary Fig. 12, three basic weaving patterns, plain, satin, and twill, are structurally designed and tested. Their corresponding voltage and current outputs were demonstrated in Fig. 3b. The textile with plain-weave pattern has the highest electrical output, followed by that of satin-weave patterned textile, while the twill-weave pattern delivers the lowest electrical output. This is mainly due to their different deformation degree under a fixed mechanical stress. Furthermore, the electricity generation process is also highly related to the mechanical excitation modes. As shown in Fig. 3c, we took the plain-woven structure textile MEG for investigation under four mechanical excitation modes, including clapping the textile with a flat surface (mode I), folding the textile parallelly (mode II) and perpendicularly (mode III) to the central magnetic fiber. In mode IV, the textile was folded along its diagonal line. As the experimental results displayed in Fig. 3d, the mode I has the best performance followed by mode II, mode III, and mode IV. This observation is also ascribed to the magnetic field variation difference in response to the four mechanical excitation modes, especially the magnetic field variation would be partially canceled in the modes II, III, and IV when the textile was folded.

To demonstrate the textile MEG as a sustainable power source, a piece of 4 cm-by-6 cm textile with plain weaving pattern was fabricated and measured. As shown in Supplementary Fig. 13, when the textile was excited by hand clapping with heavy perspiration, it delivers an ultrahigh short-circuit current of 15 mA, corresponding to a short-circuit current density of 0.63 mA cm^−2^. With the size of 4 cm by 6 cm, the textile also delivered a voltage of 4.6 V. It is worth noting that the voltage output could be greatly increased with larger size of the textile. To evaluate the output power of the textile MEG, resistors were employed as the external loads. As shown in Fig. 3e, the current decreases with the elevation of the loading resistances, while the voltage follows a reverse trend. A maximum peak power of 16 mW is obtained at the matched load of 180 Ω, corresponding to a power density of 6.67 W m^−2^ (Fig. 3f). To demonstrate the capability of the textile MEG as an intrinsically waterproof power source, the 4 cm-by-6 cm encapsulation-free textile MEG was worn on a sweaty human arm. With hand tapping, it was able to charge a 220 µF commercial capacitor to 3 V within 53 s (Fig. 3g), and drove a wearable smart biosensor system for multiple physiological information monitoring as schematically shown in Fig. 3h. Additionally, we fabricated and tested a typical triboelectric effect based biomechanical energy harvesting textile as a comparison since it has a favorable electric performance among their counterparts in the community. Under a same application scenario without encapsulation, its electrical output was adversely affected and rapidly reduced more than three order of magnitudes, as the results shown in Supplementary Fig. 14. However, the encapsulation-free textile MEG can still work properly in humid environment even immersed in water for one week (Fig. 3i). In summary, with features like intrinsic humidity-resistance, high current, and low internal resistance, the developed textile MEGs represent a milestone in the community of biomechanical energy harvesting, and could be potentially applied in medical and miniaturized robotics.

### Textile MEGs for sensing

Arterial pulse measurement is a vital means for assessment of cardiovascular disease^42^. Continuous and accurate monitoring the arterial pulse signal without influence of perspiration and fluidic environment is crucial for personalized healthcare. As a result, the ability to endure the ambient humidity is an essential property for a wearable sensor.

**Fig. 4:**
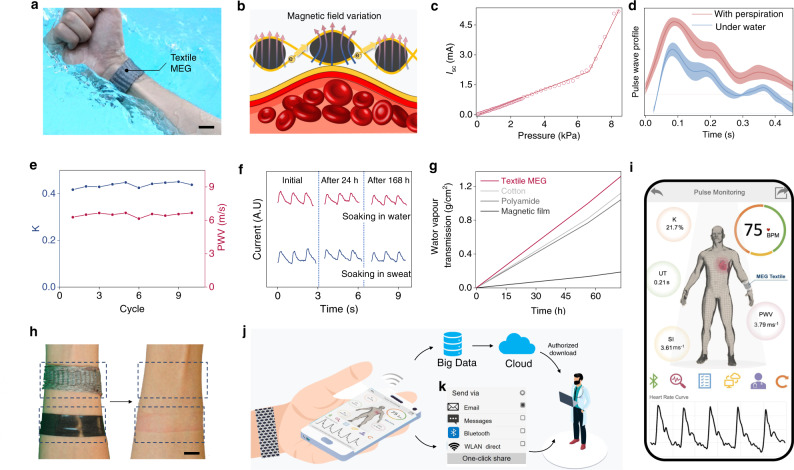
Measuring cardiovascular parameters underwater without encapsulation for telehealth. **a** Photograph of the textile wristband as a wearable pulse sensor. Scale bar: 2 cm. **b** Schematics of the working principle of the textile wristband. **c** Sensitivity of the textile MEG with respect to applied pressure at a frequency of 1 Hz. **d** Measured pulse wave profile in one cardiac cycle when the encapsulation-free textile wristband was tested with perspiration and under water. The shadow areas indicate the standard deviation of the results from five pulse wave profiles. Data are mean ± SD; *n* = 5. **e** Characteristic *K* value and PWV obtained from pulse wave signals. **f** Measured pulse wave profiles when textile wristband was soaked in actual sweat and water for up to 168 h. The pulse wave in blue color was tested in actual perspiration status. **g** A water vapor transmission rate test showing the perspiration transmission through the textile MEG. **h** Photograph showing the skin irritation results of the permeable textile MEG and an impermeable magnetic film on the forearm. Scale bar: 1.5 cm. **i** The developed user interface on a cellphone APP. **j** Schematic of a fully integrated CMS. The personal health data could be processed via two pathways: directly sending to the physicians for immediate clinical diagnosis, or uploading into the cloud to conduct big data analysis for data-driven diagnosis. **k** Screenshot of mobile user interface showing the personal health data sharing options.

With fundamentally new working principle, the textile MEG is a promising candidate in continuous arterial pulse monitoring because it can operate steady in humid environment without compromising its electrical performance and wearability. To demonstrate the textile MEG as an encapsulation-free and intrinsically waterproof pulse wave sensor for continuous cardiovascular parameters measurement, the textile MEG was fabricated into a wristband and worn against the wrist artery in an underwater situation (Fig. 4a). Pulsation of blood vessels induce soft magnetic fiber deformation, which will sensitively cause the magnetic field variation within the soft fiber due to the strong magnetomechanical coupling. As a consequence, the tiny arterial pressure fluctuations are converted into high-quality electrical signals via the textile wristband for further cardiovascular system characterization (Fig. 4b). To study the sensitivity of the textile MEG, loading–unloading tests were conducted at 1 Hz with increased applied pressure. As shown in Fig. 4c, the textile MEG can detect pressure as low as 0.05 kPa. The current responses linearly with the increased pressure in the range from 0.1 to 6.52 kPa, and then the current increase quicker in the high-pressure range, which may be due to the non-linearity of magnetoelastic effect in the high-pressure range.

The generated pulse waveforms via arterial pressure fluctuations were collected by textile wristband and presented in Fig. 4d, in which the shadow areas indicate the standard deviation. Three characteristic points of the arterial pulse wave including systolic, reflected, and diastolic peaks are accurately expressed by the electrical signals no matter tested with perspiration or under water (Supplementary Fig. 15). It is worth noting that the pulse waveform is affected by different contact tightness between the textile wristband and the skin, and pulse signal can be clearly obtained with 2 kPa pressure (Supplementary Fig. 16). Apart from this, when the textile MEG tested under a large pre-pressure up to 392 kPa, it can still detect pressure fluctuations, which will be favorable for deep water applications (Supplementary Fig. 17). We took a further step to perform the cardiovascular parameters analysis. Heart rates (HR) are derived from peak position extracted from detected pulse waveform. The characteristic *K* value indicates the mean arterial blood pressure and the pulse wave velocity (PWV) reflects the elasticity and compliance of artery, which were plotted in the Fig. 4e. The stiff index (SI) is sensitive to the artery stiffness, and the upstroke time (UT) represents the ascending time of the systolic stage^43^, which were also presented in the Supplementary Fig. 18a. These five characteristic parameters HR, *K*, PWV, SI, and UT, are highly correlated with pathological changes. Detailed calculations are presented in Supplementary Note 3. Furthermore, both the mechanical stability against deformation and chemical stability against sweat corrosion are of great importance to the textile wristband for long-term continuous biomonitoring. On the one hand, loading and unloading mechanical deformation up to 20,000 times was applied to the textile wristband. No obvious decay was observed from the electrical output (Supplementary Fig. 18b). On the other hand, artificial perspiration was employed to test its chemical stability. As the results presented in Fig. 4f, the encapsulation-free textile wristband still can accurately detect the pulse waveforms after being immersed in water or perspiration for up to 168 h. In order to demonstrate its stability, a longer period pulse wave and heart rate measurement for 2 h were demonstrated in Supplementary Fig. 19. These experimental observations fully demonstrated that the textile MEG is corrosion resistant and durable, owing to the stable remanent magnetization and large coercive field of nanomagnets (Supplementary Fig. 20)^35^.

Furthermore, the wearability of the textile MEG is also an important factor for long-term cardiovascular monitoring, so we further tested its permeability. As shown in Fig. 4g, the textile MEG has the highest water vapor transmission rate of 0.018 g cm^−2^ h^−1^, which is higher than cotton, polyamide film, and magnetic film (Supplementary Table 3). This is due to the porous structure design of the textile and nanoscale-to-microscale cavities in the soft magnetic fibers. Then, an on-skin test was done to prove its wearability. As shown in Fig. 4h, textile MEG did not caused negative effect on the skin after wearing for 1 week. However, the impermeable magnetic film caused skin irritation. This is mainly due to the perspiration can pass through textile MEG quickly (Supplementary Fig. 21 and Supplementary Movie 2) and water vapor can easily escape to the ambient environment, which ensures the wearer’s comfort. Furthermore, the magnetic fiber was confirmed to be biocompatible as verified by in vitro culture of mouse fibroblasts. As shown in Supplementary Fig. 22a and 22b, the cells on microfibers exhibit regular morphology with rare cell death, similar to the cells in tissue culture dish (control), whereas almost all the cells died in the negative control group treated with 20% dimethylsulfoxide (DMSO) (Supplementary Fig. 22c). We also quantitatively assessed the cell viability. Over 97.6% of mouse fibroblasts on magnetic fibers survived after 24 h, indicating the excellent biocompatibility of magnetic fibers (Supplementary Fig. 22d).

For user-friendliness toward practical application, a customized mobile APP with built-in algorithm was developed and integrated into the textile wristband to form a wireless cardiovascular monitoring system (CMS) for continuous measurement. As the system-level block diagram illustrated in Supplementary Fig. 23, the CMS begins with pulse signal acquisition via the textile wristband; after signal conditioning and data processing, pules wave signals were wirelessly transmitted to a cellphone with a Bluetooth module. With the built-in algorithm, the wearable CMS is able to analyze the real-time pulse wave signals, measure the characteristic parameters including HR, K, PWV, SI, and UT, and timely display the health data on the cellphone APP (Fig. 4i). In order to present a comprehensive evaluation of individual cardiovascular system, five sets of curves were continuously displayed to track the dynamic change of characteristic parameters HR, K, PWV, SI, and UT, as the screenshot of the cellphone APP demonstrated in Supplementary Fig. 24. As illustrated in Fig. 4j, in our designed wireless CMS, the generated personal health data could be further processed via two pathways. One is directly sent to the physicians for immediate clinical diagnosis. Through a simple one-click, the datasets could be sent to physicians via email, message, Bluetooth, or wireless local area network (WLAN) direct, as the screenshots of the App shown in Fig. 4k and Supplementary Fig. 25. The other pathway is to upload the measured health data into the cloud to build up personal health database for big data analysis, which is available to the physician for data-driven diagnosis via authorized downloading. In order to visualize the operational features of the developed wireless CMS, a movie was recorded to demonstrate the wearable cardiovascular system management in the heavily sweaty situation (Supplementary Movie 3). In summary, the intrinsically waterproof textile MEG enabled wireless CMS is providing a comfort and comprehensive solution for continuous cardiovascular system management. It represents a practical step to build up soft-matter technology towards telemedicine for personalized healthcare in the era of the Internet of things.

## Discussion

The magnetoelastic effect can convert tiny pressure to enormous magnetic field variation via altering the magnetic dipole interaction in a fiber. To explore the strong magnetomechanical coupling in the soft system, a textile MEG was developed for high-performance biomechanics-induced electricity generation. Compared to other existing textile counterparts, the textile MEG distinguishes itself in many aspects, including intrinsic humidity-resistance, high current, and low internal resistance.

From a fundamental science perspective, the textile MEG is built on a two-step conversion mechanism coupling magnetoelastic effect and magnetic induction. The observed magnetoelastic effect in the soft fiber assures the highly efficient mechanical-to-magnetic conversion. It distinguishes from magnetoelastic metal alloys in larger magnetomechanical coefficient, lower Young’s modulus, and no required magnetic field, as summarized in Supplementary Table 1. With a following step of magnetic-to-electrical conversion, the textile MEG exhibits a short-circuit current density of 0.63 mA cm^−2^ and an internal impedance of 180 Ω. Our textile MEG is mainly based on mechanical force-induced magnetic dipole realignment in the soft fibers. Technically, the power density of the textile MEG can be further improved by weaving multilayer conductive yarns and soft magnetic fibers into a 3D form, as illustrated in Supplementary Fig. 26. We envision that the textile MEGs with versatile configurations represent a novel platform of soft-matter electronics for energy, medical care, miniaturized robotics, and artificial perception.

We are convinced that the observed magnetoelastic effect in soft fibers is not restricted to being coupled with magnetic induction for electricity generation as we demonstrated here. We foresee that it could be widely adopted to establish various soft-matter technologies and open doors to many other fields. For instance, when it is coupled with magnetocaloric effect, the thermodynamics of materials could be controlled with applied stress to present a mechanical force driven active personal thermoregulation. When it is coupled with magneto-optic effects, the refractive index of a material could be altered in response to applied stress to invent various soft matter systems for mechanical force induced optical regulation for controlling the transmission, reflection, and absorption of light.

In summary, we observed the magnetoelastic effect in soft fibers that shows higher magnetomechanical coupling than traditional magnetoelastic effect in metal alloys. Based on the fibers, a textile MEG is constructed as an emerging approach for intrinsically waterproof wearable biomechanical energy conversion. With heavy perspiration, the textile MEG was demonstrated to deliver a short-circuit current density of 0.63 mA cm^−2^ with an internal impedance of 180 Ω. A textile wristband was also developed to convert the arterial pulse wave into high-quality electrical signals in an underwater scenario. A customized mobile APP with built-in algorithm was integrated into the textile wristband to form a wireless CMS, which could provide telemedicine via both immediate clinical and data-driven diagnosis. With features like intrinsic humidity-resistance, high current, and low internal resistance, we envision textile MEGs with adaptable forms be widely adopted to build up versatile soft matter electronics. Moreover, it is worth noting that the magnetoelastic effect in 1D soft fibers could also be further coupled with magneto-optic effect and magnetocaloric effect to invent compelling soft-matter technologies and open doors to pressure induced thermal and optical regulations.

## Methods

### Fabrication of 1D soft magnetitic fiber

The neodymium–iron–boron (NdFeB) nanomagnets (Magnequench) were firstly coated with a layer of SiO_2_ by hydrolysis and polycondensation of tetraethyl orthosilicate (Sigma Aldrich). Subsequently, NdFeB nanomagnets were mixed with Ecoflex 00-30 part A (Smooth-on Inc.). Then, the Ecoflex 00-30 part B (Smooth-on Inc.) was added to the mixture with the same weight as part A. The weight percent of the NdFeB nanomagnets varies from 50, 75, and 83 wt% with the silicone polymer. After mixing thoroughly for 10 min to introduce microbubbles, the three-phase mixture was added to a syringe. Then, the syringe was put into a customized mechanical platform, in which the plunger was propelled by a linear motor (LinMot, H01-37). The soft magnetic fiber was extruded out under pressure of ~300 kPa via a nozzle. The extruded fiber was collected with a metal plate, which was then placed on a hotplate at 60 °C for 30 min (Fisher Scientific, Isotemp). Subsequently, the soft magnetic fiber was magnetized at impulse fields (2.65 T) by an impulse magnetizer (IM-10-30, ASC Scientific) with programmed directions. Soft magnetic fibers made by 83 wt% of SrFe_12_O_19_ (Sigma Aldrich) or Fe_3_O_4_ (Sigma Aldrich) are also fabricated with the same procedure as NdFeB.

### Fabrication of a conductive yarn

First, the nylon microfibers (Systcom Advanced Materials Inc.) were cleaned with diethyl ether (Sigma Aldrich) solution, then they were immersed in a solution mixed by HCl (Sigma Aldrich, 37%) 5 mL/L and SnCl_2_ (Sigma Aldrich) 5 g/L for 10 min. After that, the microfibers were placed into one liter of solution A, which consists of AgNO_3_ (Alfa Aesar) 12 g/L, NaOH (Sigma Aldrich) 8 g/L, NH_4_OH (Sigma Aldrich, 37%) 200 mL/L. Then, 3 L of glucose (Alfa Aesar) solution (2.6 g/L) were poured in the solution A to perform the glucose reduction reaction. Finally, the microfibers were taken out and cleaned with deionized water for three times. Subsequently, the silver-coated nylon microfibers were intertwined by a braiding machine (Juzheng Industry Co., Ltd., JZ0011) to a conductive yarn.

### Textile fabrication by a weaving loom

The textile MEG was fabricated by a desktop weaving loom (Arpakasso, zbj-02) via a shuttle flying process (Supplementary Movie 4). The stress intension of the string was kept constant in the weaving process by a specially designed pully system. For the vertical weaving pattern, the soft magnetic fibers were employed as the warp yarn, the conductive yarns were inserted as the filling yarn. To obtain the parallel weaving pattern, nylon wires were employed as the warp yarn, the conductive yarn and soft magnetic fibers were inserted as the filling yarn. For the textile MEG used in CMS, silver-coated yarns were employed as conductive yarn, and the textile MEG is fabricated with size of 3 cm by 13 cm. For the biomechanical energy harvesting application, copper wires were employed as conductive yarn. To charge capacitors, the textile MEG is fabricated with size of 4 cm by 6 cm using conductive yarns and soft magnetic fibers with cross section area of 12 mm^2^. Moreover, wool fibers were also woven into the textile to improve the textile wearability. It is worth noting that both the fibers fabrication and textile weaving process are straightforward and compatible with massive production.

### Preparation of the textile based on triboelectric effect

Copper foil (thickness of 50 µm) was sandwiched between two fluorinated ethylene propylene (FEP) layers. Then they are cut into stripes with a width with 0.5 mm via a laser cutting machine (ULTRA R5000, Universal Laser System). After the FEP was weaved with copper stripe with plain pattern, the copper stripe was connected with copper wire to export the electric output.

### Water vapor transmission rate test

The test was based on ASTM E94 with modification. Glass bottles were filled with deionized water and sealed by tested textile samples. The glass bottles were placed in an environment of 35 °C and relative humidity at ~30%. The mass of the bottles was weighted by an electronic balance after 72.5 h. By calculated the reduced mass of the bottles divided by area of textiles, the water vapor transmission rate was obtained.

### Preparation of artificial perspiration

The artificial perspiration was used to test the chemical stability of the textile wristband. To prepare it, 4.65 g NaCl (Sigma Aldrich), 3.87 g 1 M lactic acid solution (Alfa Aesar), 1.80 g Urea (Alfa Aesar), 1.37 g KCl (Sigma Aldrich), 0.756 g NaHCO_3_ (Sigma Aldrich), 0.546 g 1 M NH_3_·H_2_O (Sigma Aldrich), 0.175 g Na_2_SO_4_ (Sigma Aldrich), and 0.0276 g uric acid (Alfa Aesar) were added in 3 L deionized water and mixed for 30 min.

### Structure characterization

The morphology of the soft magnetic fiber and conductive yarn were characterized by scanning electron microscopy (Zeiss, supra 40VP). The micro-computed tomography (Micro-CT) image of the magnetic fiber was scanned at 80 kVp/140 μA with 500 ms exposure using a μCT scanner (HiCT) developed by the Crump Institute for Molecular Imaging at UCLA. The 2D magnetic flux density mapping on the surface of the soft magnetic fiber was achieved by continuously measuring the magnetic field using the digital Gauss meter mounted on a two axial motion platform. The stress–strain curves were tested by a dynamic mechanical analyzer (DMA, RSA III). First, the dog bone specimens with different magnetic concentration (50%, 75%, and 83%) were clamped at the mechanical analyzer; then the curves were obtained at a fixed distance of 5 mm using the tensile testing at a stretching rate of 0.25 mm s^−1^. Young’s modulus was calculated via fitting the tested curves with a Neo-Hookean model. Magnetic hysteresis loop was tested by a SQUID magnetometer (Quantum Design, MPMS3).

### Electrical performance measurement

The voltage and current signals of the textile MEG were measured by the electrometer (6514, Keithley). A flat plate larger than the size of the textile were used to replace human hands for standard test. The flat plate was connected with an electrodynamic shaker system, which consists of a function generator (AFG1062, Newark), a linear power amplifier (PA-151, Labworks Inc.), and an electrodynamic transducer (ET-126HF, Labworks Inc.). To charge the capacitors, the generated electricity was processed with a diode bridge rectifier (MBSK16SE) and a toroidal transformer.

### Design the circuitry and user interface for wireless CMS

A customized printed circuit board (PCB) was designed to acquire the pulse wave signals from textile wristband. The whole device was waterproof by coating a thin transparent hydrophobic layer on the circuit produced by Auland Technology Co., Ltd. The PCB has three components. First, an analog circuit was used for pulse signal conditioning. Next, a micro-controller unit (MCU, Arduino Nano) was used to collect and convert the analog pulse signals to digital pulse signal, and thirdly the onboard Bluetooth module was used to wirelessly transmit the digital data to cellphone. The cellphone APP was created via MIT AI2 Companion. The heart rate, PWV, stiffness index, *K* value, and UT were calculated with the assistance of the peak detection algorithms.

### Human subject study

The textile wristband used for wearable cardiovascular monitoring was performed using human subjects in compliance with all the ethical regulations under a protocol (ID: 20-001882) that was approved by the Institutional Review Board (IRB) at University of California, Los Angeles. All participating subjects belonged to University of California, Los Angeles and were provided informed consent before participation in the study.

### Biocompatibility test of soft magnetic fiber

Mouse fibroblasts were isolated from the mouse ear and expanded in fibroblast medium by following the protocol approved by the Institutional Animal Care and Use Committee of University of California, Los Angeles (protocol ARC-2016-101). These fibroblasts were cultured in an incubator at 37 °C and 5% CO_2_. For cell viability assay, before seeding cells onto magnetic fibers, magnetic fibers were plasma-treated for 1 min and coated with 0.1% gelatin for 1 h. Then fibroblasts were plated and allowed to attach to the magnetic fibers for 24 h. The cell viability was quantified by using the Live/Dead assay and PrestoBlue^®^ Cell Viability Reagent (Invitrogen, A13261) according to the manufacturer’s protocol. Cells were incubated with the 10% PrestoBlue reagent for 2 h. Results were normalized to control samples (i.e., cells seeded in tissue culture plate). Live/Dead assays were performed by using the LIVE/DEAD^TM^ Cell Imaging Kit (Invitrogen, R37601) according to the manufacturer’s protocol. Cells were incubated with an equal volume of 2× working solution for 30 min at room temperature. Epifluorescence images were collected by using a Zeiss Axio Observer Z1 inverted fluorescence microscope and analyzed using Image J software. In addition, cells were incubated with the 10% PrestoBlue Reagent for 2 h. Absorbance was measured by a plate reader (Infinite 200PRO) at excitation/emission = 560/590 nm. Results were normalized to control samples (i.e., cells seeded in tissue culture dish).

### Statistics and reproducibility

In all the experiments, we repeat the test for five times independently with similar results.

## Supplementary information

Supplementary Information

Description of Additional Supplementary Files

Supplementary Movie 1

Supplementary Movie 2

Supplementary Movie 3

Supplementary Movie 4

## Data Availability

Source data are provided with this paper. Other data generated or analysed during this study are included in the Supplementary Information. Source data are provided with this paper.

## Source data

Source Data
