# Supplementary material for: Soft fibers with magnetoelasticity for wearable electronics
Source: Nat Commun. 2021 Nov 19;12:6755. doi: 10.1038/s41467-021-27066-1 (PMC8604991; doi:10.1038/s41467-021-27066-1)
Supplement: Supplementary file 1 — Supplementary Information [file 41467_2021_27066_MOESM1_ESM.pdf]

Supplementary Information

Soft fibers with magnetoelasticity for wearable electronics

Xun Zhao[1,#], Yihao Zhou[1,#], Jing Xu[1], Guorui Chen[1], Yunsheng Fang[1], Trinny Tat[1], Xiao Xiao[1], Yang Song[1], Song Li[1], Jun Chen[1,*]

[1]Department of Bioengineering, University of California, Los Angeles, Los Angeles, CA 90095, USA

[#]These authors contributed equally to this work.

* Correspondence to jun.chen@ucla.edu (J.C.)

This PDF file includes:

Supplementary Figures 1-29

Supplementary Notes 1-4

Supplementary Tables 1-3

Supplementary References

Table of Contents

Supplementary Figure 1. Scanning electron microscope (SEM) image of soft magnetic fiber. 3
Supplementary Figure 2. Characterization of the soft magnetic fibers via Micro-CT. 4
Supplementary Figure 3. Stress strain curves of the soft magnetic fibers. 5
Supplementary Figure 4. Photograph of three spools of magnetic fibers. 6
Supplementary Figure 5. Schematics showing a two-axial motion platform. 7
Supplementary Figure 6. Magnetic flux density mapping of the soft magnetic fiber. 8
Supplementary Figure 7. Magnetic flux density mapping of the soft magnetic fiber. 9
Supplementary Figure 8. Investigation of the magnetic flux density variation. 10
Supplementary Figure 9. Comparison of the experimental magnetic field variation and the wavy chain model. 11
Supplementary Figure 10. Massive production of the conductive yarns. 12
Supplementary Figure 11. SEM image of the silver-coated nylon microfibers. 13
Supplementary Figure 12. Schematics showing three different weaving patterns. 14
Supplementary Figure 13. Electrical output of textile MEG under continuous hand tapping. 15
Supplementary Figure 14. The schematics of textile based on triboelectric effect. 16
Supplementary Figure 15. Photographs of the textile. 17
Supplementary Figure 16. The generated pulse wave from textile wristband. 18
Supplementary Figure 17. Current output of the sensor with pre-loading of 0 Pa and 392 kPa. 19
Supplementary Figure 18. Measuring cardiovascular parameters with the textile wristband 20
Supplementary Figure 19. The generated pulse wave and heartbeat after textile wristband 21
Supplementary Figure 20. Magnetic hysteresis loop of the NdFeB nanomagnets. 22
Supplementary Figure 21. Photographs of the perspiration droplet passing through textile MEG. 23
Supplementary Figure 22. Biocompatibility of magnetic fiber 24
Supplementary Figure 23. System-level block diagram of the wireless wearable CMS. 25
Supplementary Figure 24. Screenshot of the cellphone App. 26
Supplementary Figure 25. Schematics of the cellphone App. 27
Supplementary Figure 26. Schematics of the 3D MEG textiles. 28
Supplementary Note 1. Detailed comparison of the magnetorheological elastomers. 29
Supplementary Note 2. Theoretical explanation of the wavy chain analytical model. 31
Supplementary Note 3. Calculation of energy conversion efficiency. 33
Supplementary Note 4. Cardiovascular parameters analysis. 34
Supplementary Table 1. Comparison of four magnetoelastic materials and textile MEG. 36
Supplementary Table 2. Price of the magnetic fiber and conductive yarn. 37
Supplementary Table 3. Water vapor transmission rate of different textile samples. 38
Supplementary References 39

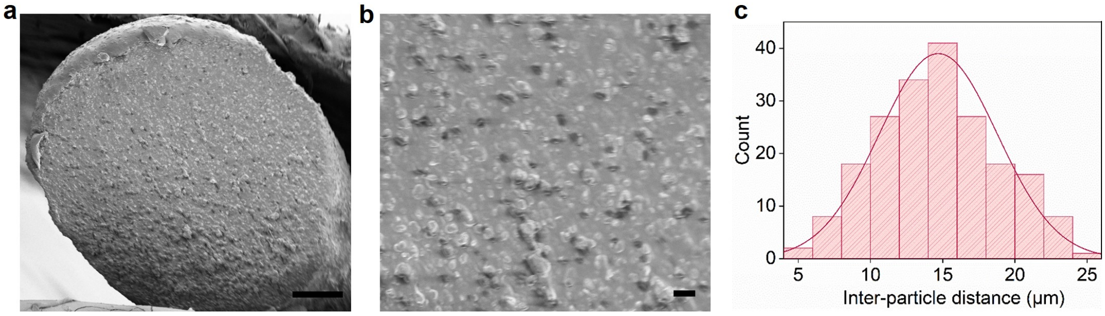

Supplementary Figure 1. Scanning electron microscope (SEM) image of cross-section view of a soft magnetic fiber and inter-particle distance histogram in the soft magnetic fiber. a, Scale bar: 100 µm. **b**, Scale bar, 20 µm. **c**, Inter-particle distance histogram derived from the cross-section-view SEM images of the magnetic fiber.

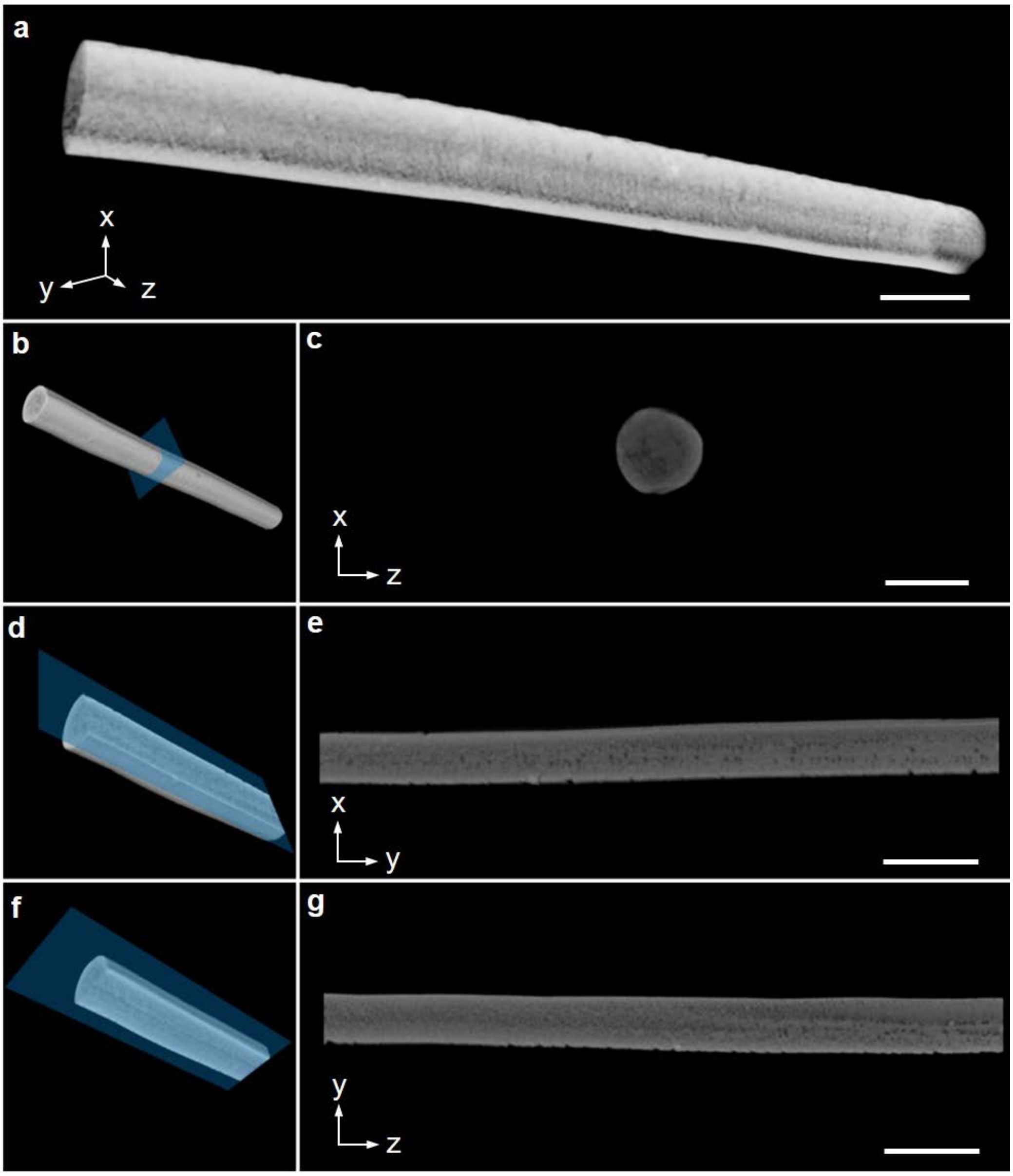

Supplementary Figure 2. Characterization of the soft magnetic fibers via Micro-CT image. a, 3D Micro-CT image showing the whole view of the magnetic fiber. Scale bar: 1.5 mm. **b**, Schematics and **c**, Micro-CT image showing the XZ section view of the magnetic fiber. Scale bar: 1.5 mm. **d**, Schematics and **e**, Micro-CT image showing the XY section view of the magnetic fiber. Scale bar: 2 mm. **f**, Schematics and **g**, Micro-CT image showing the YZ section view of the magnetic fiber. Scale bar: 2 mm.

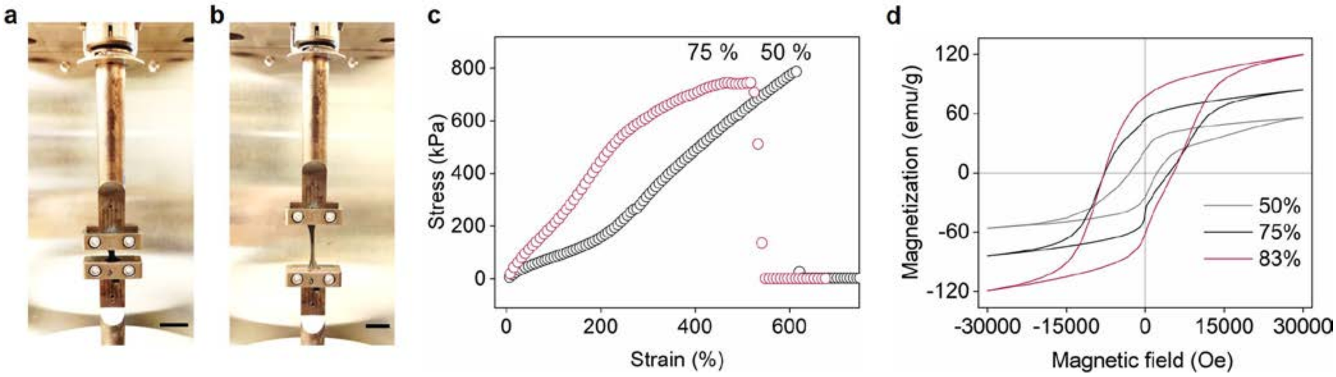

Supplementary Figure 3. Stress strain curves of the soft magnetic fibers. a-b, The photography of the soft magnetic fibers: **a,** In the initial state. **b,** In the stretching state. Scale bars: 1 cm. **c,** Stress strain curves of the soft magnetic fibers with 75 wt% and 50 wt% magnetic concentrations. **d,** Magnetic hysteresis loop of the soft magnetic fibers with 83 wt%, 75 wt% and 50 wt% magnetic concentrations.

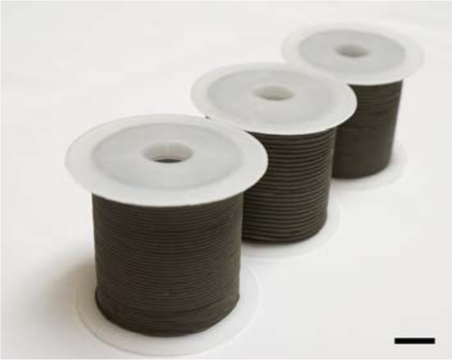

Supplementary Figure 4. Photograph of three spools of magnetic fibers. Scale bar: 1.4 cm.

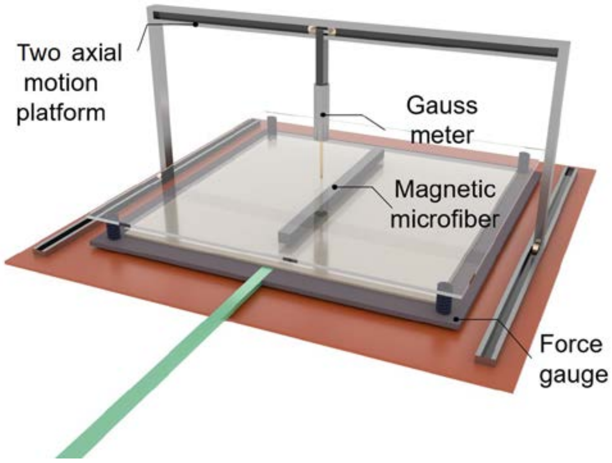

Supplementary Figure 5. Schematics showing a two-axial motion platform.

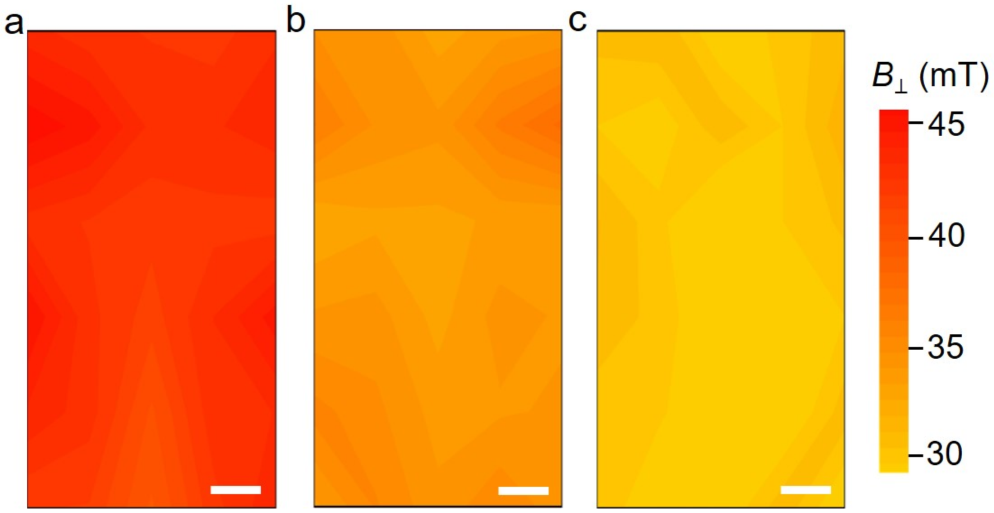

Supplementary Figure 6. Magnetic flux density mapping of the soft magnetic fiber. a-c, Magnetic flux density mapping of the soft magnetic fiber on north pole surface in the original state (**a**), under a compressed pressure 0 kPa (**b**) 180 kPa and (**c**) 300 kPa. Scale bars: 0.24 mm.

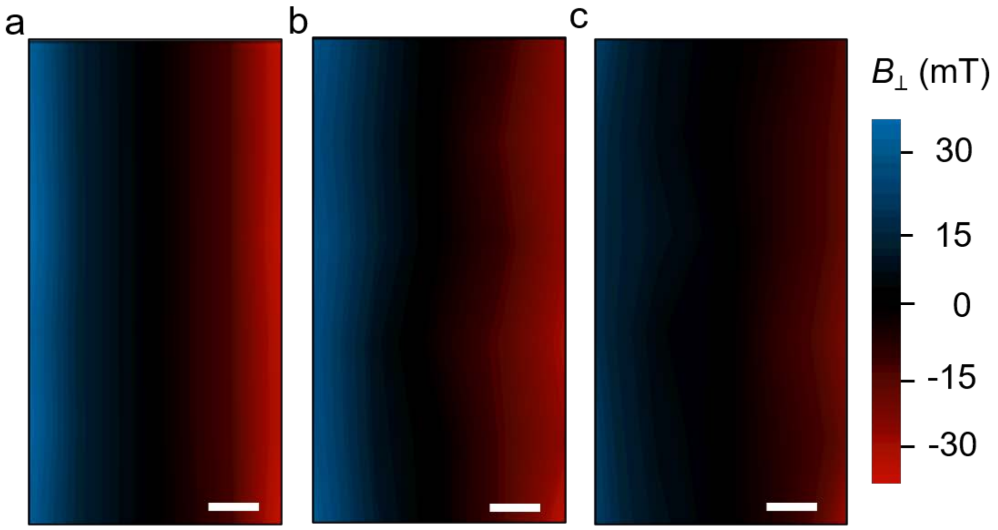

Supplementary Figure 7. Magnetic flux density mapping of the soft magnetic fiber. a-c, Magnetic flux density mapping of the soft magnetic fiber on side surface in the original state (**a**) under a compressed pressure 0 kPa (**b**) 40 kPa and (**c**) 86 kPa. Scale bars: 0.24 mm.

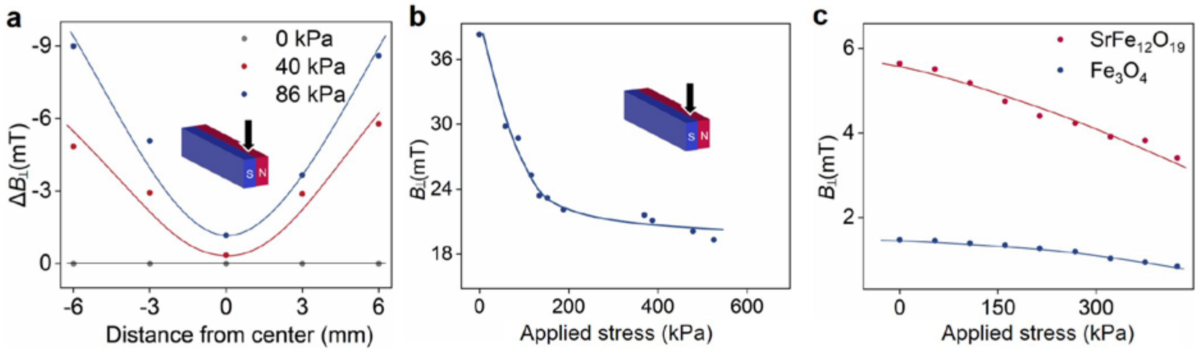

Supplementary Figure 8. Investigation of the magnetic flux density variation of the soft magnetic fiber in response to the applied compressive pressure. a, Magnetic field variation of the soft magnetic fiber under applied stress on the side surface. **b**, Magnetic field reduction of the soft magnetic fiber under applied stress on side surface. **c**, Magnetic field reduction of the soft magnetic fibers with 83 wt% of $SrFe_{12}O_{19}$ or Fe_3O_4 under applied stress.

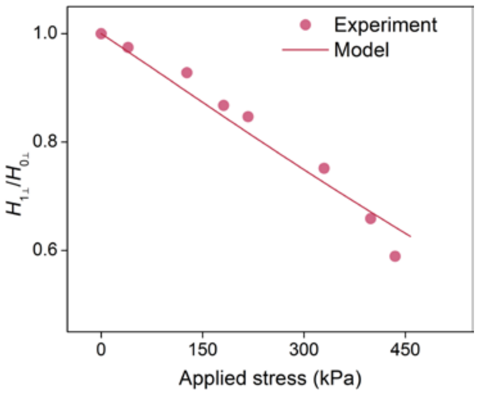

Supplementary Figure 9. Comparison of the experimental magnetic field variation and the wavy chain model prediction showing consistency.

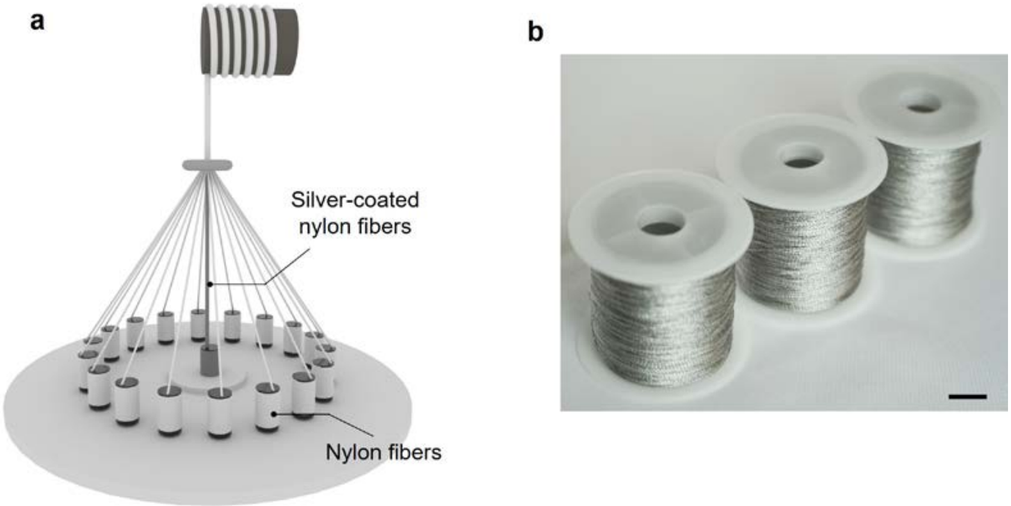

Supplementary Figure 10. Massive production of the conductive yarns. a, Schematics showing braiding machine. **b,** Photograph of three spools of conductive yarns. Scale bar: 1.3 cm.

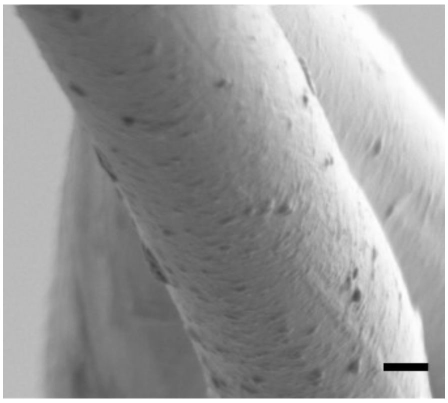

Supplementary Figure 11. SEM image of the silver-coated nylon microfibers. Scale bar: 4 µm.

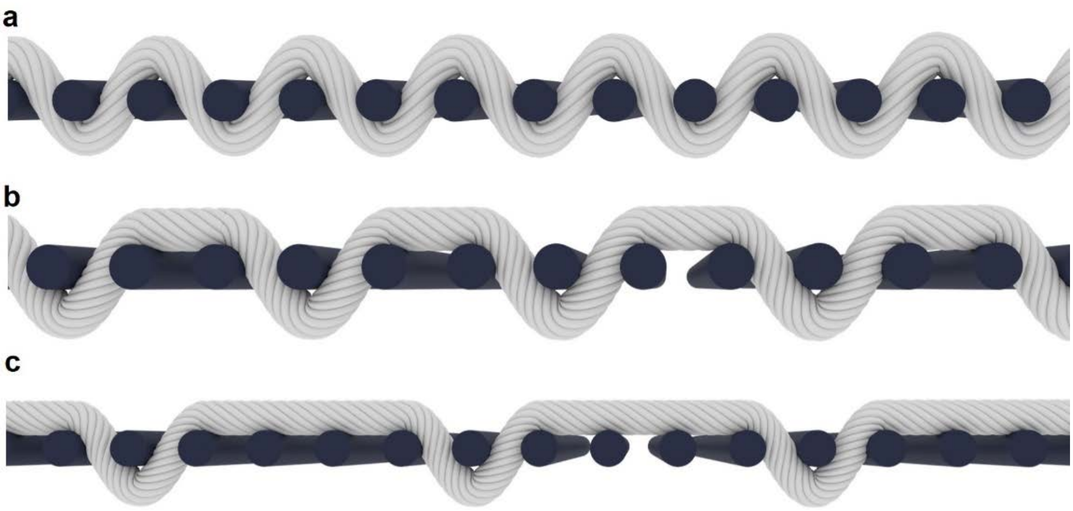

Supplementary Figure 12. Schematics showing three different weaving patterns. a, Plain. **b**, Satin. **c**, Twill.

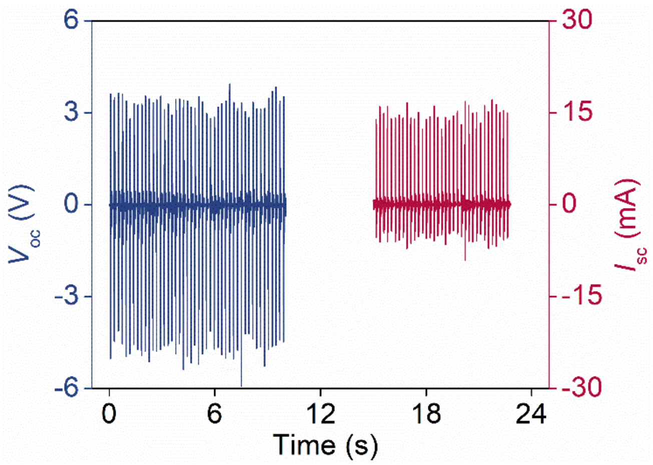

Supplementary Figure 13. Electrical output of the textile MEG under continuous hand tapping.

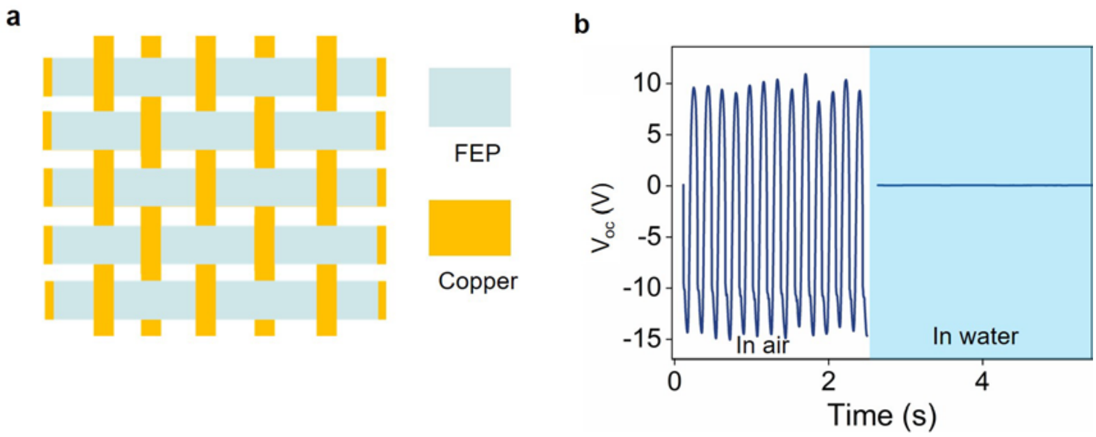

Supplementary Figure 14. The schematics and electrical performance of textile based on triboelectric effect. a, Schematics of textile based on triboelectric effect. **b**, Voltage output of textile based on triboelectric effect testing in air and in water condition.

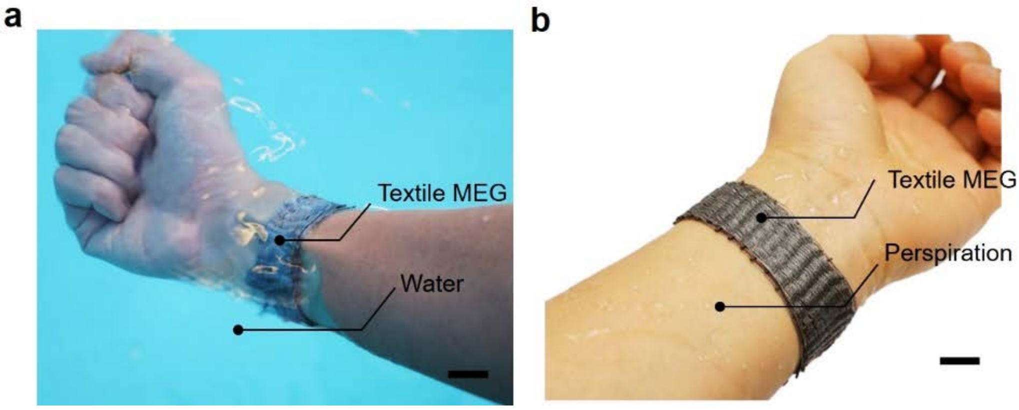

Supplementary Figure 15. Photographs of the textile wristband. a, Textile wristband tested under water. Scale bar: 1.5 cm. **b**, Textile wristband tested with artificial perspiration condition. Scale bar: 1.5 cm.

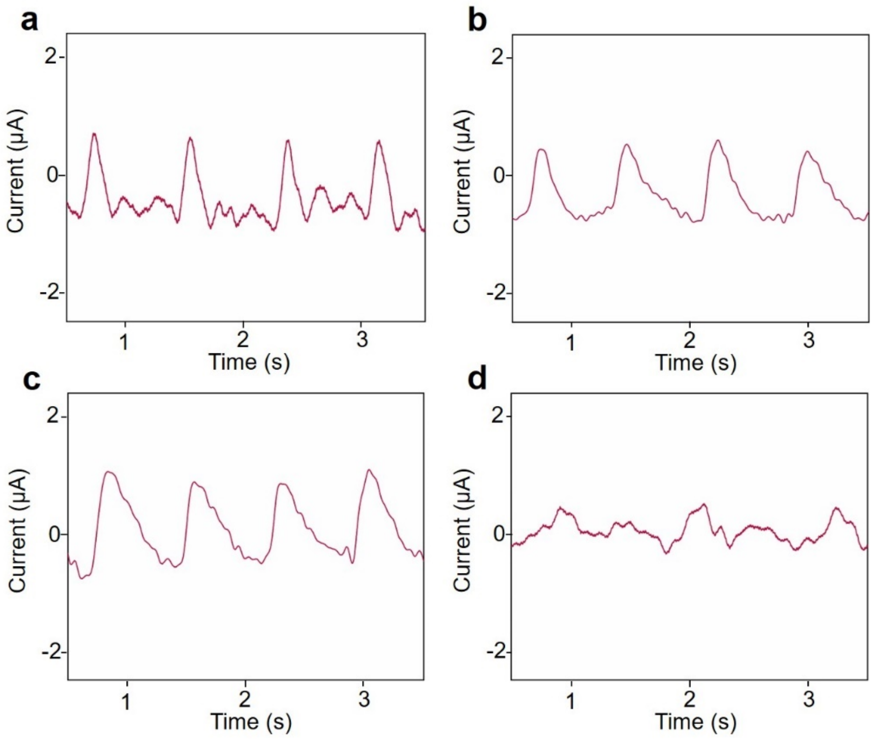

Supplementary Figure 16. The generated pulse wave from textile wristband with different tightness between the textile wristband and the skin. a, 15 Pa. **b,** 0.5 kPa. **c**, 2 kPa. **d**, 50 kPa.

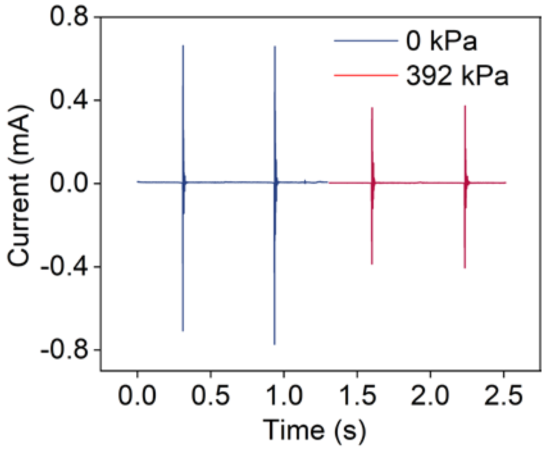

Supplementary Figure 17. Current output of the sensor responding to a movable substrate with pre-loading of 0 kPa and 392 kPa.

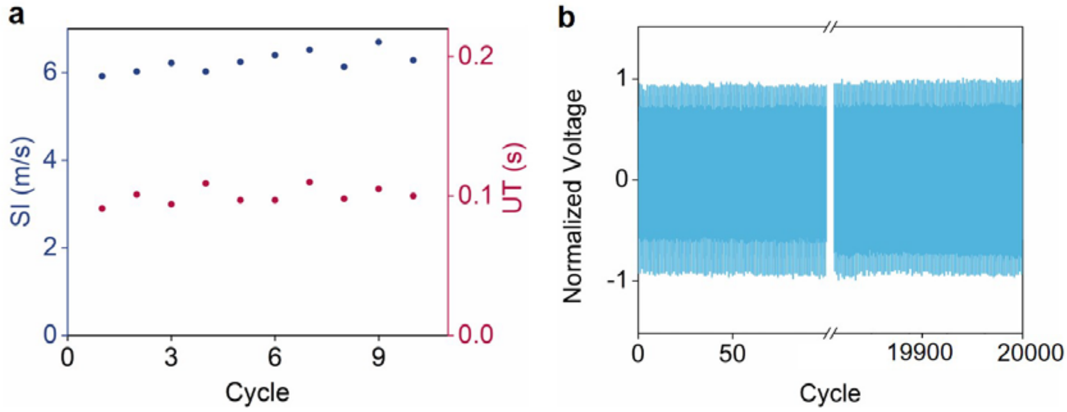

Supplementary Figure 18. Measuring cardiovascular parameters with the textile wristband. a, Characteristic SI, UT obtained from pulse wave profiles. **b**, A loading and unloading test were performed for 20,000 times.

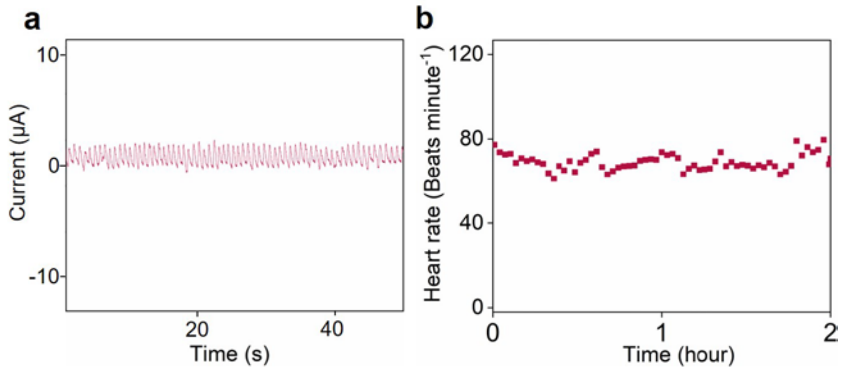

Supplementary Figure 19. The generated pulse wave and heartbeat after textile wristband was soaked in water for up to 168 hours. a, The generated pulse collected by textile wristband. **b,** Testing heart rate trends by textile wristband for two hours.

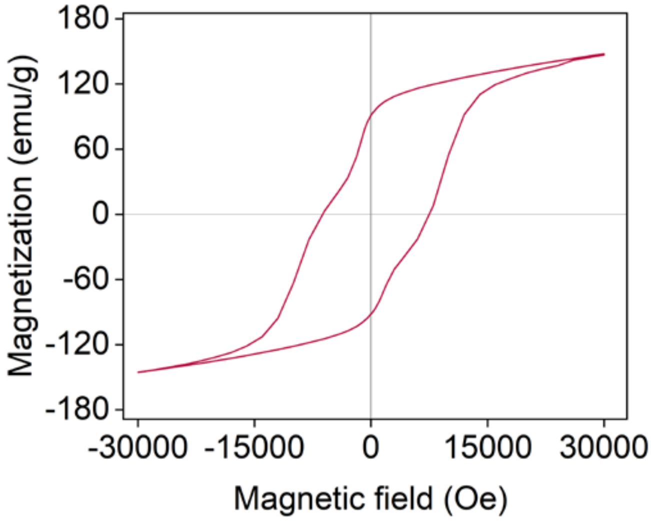

Supplementary Figure 20. Magnetic hysteresis loop of the NdFeB nanomagnets.

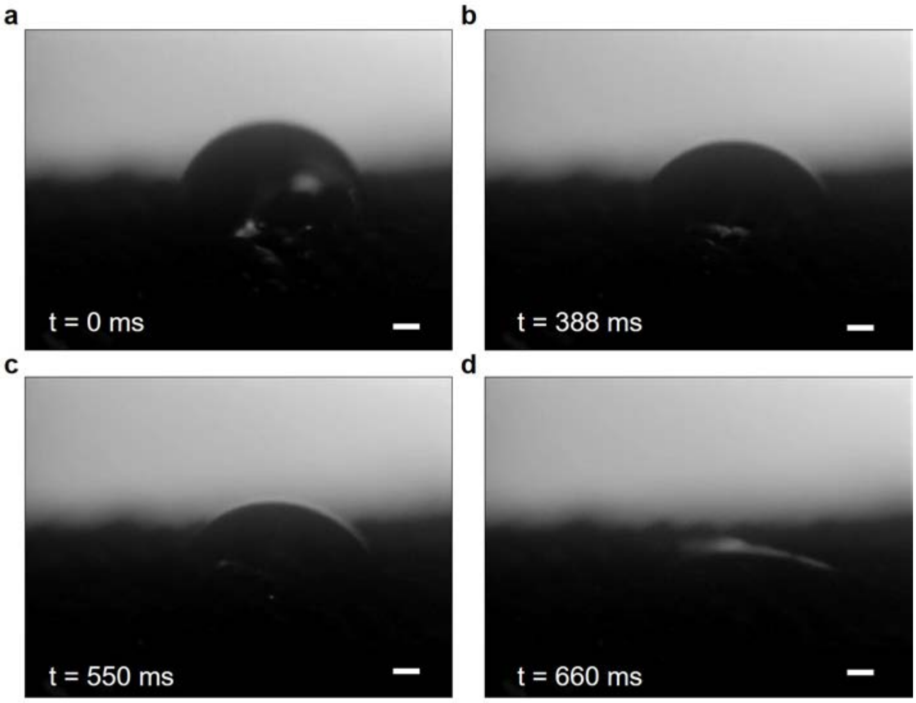

Supplementary Figure 21. a-d, Photographs of the artificial perspiration droplet passing through textile MEG: (**a**) 0 ms (**b**) 388 ms (**c**) 550 ms and (**d**) 660 ms. Scale bars: 0.5 mm.

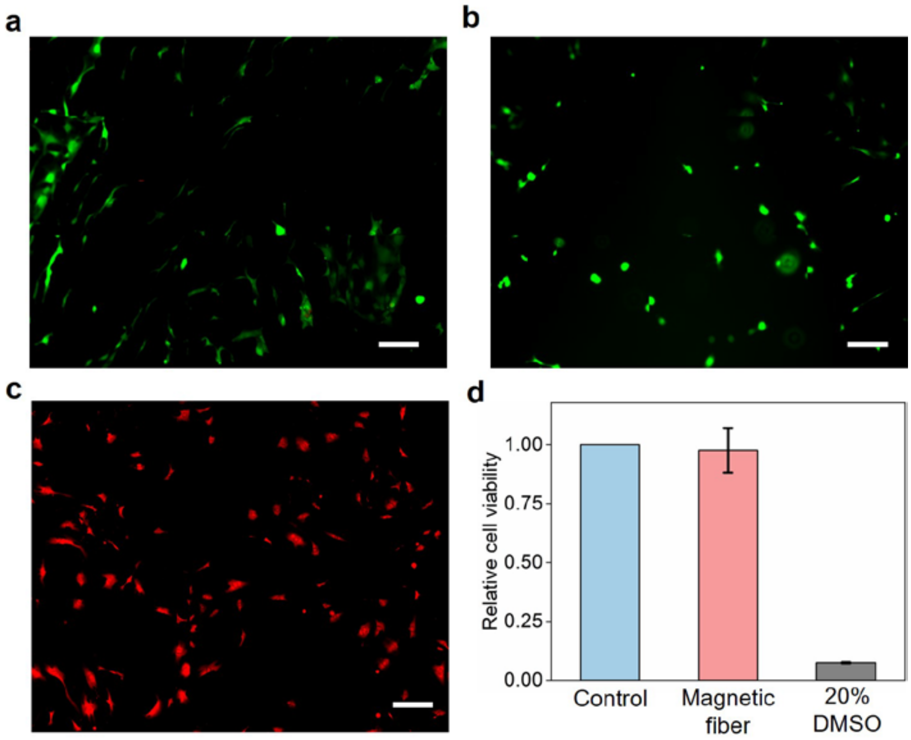

Supplementary Figure 22. Biocompatibility of magnetic fiber. (a-c) Mouse fibroblasts were grown in tissue culture dish as a control (**a**), cultured on magnetic fiber (**b**), or treated with 20% DMSO (negative control) (**c**) for 24 hours, followed by Live/Dead assay and fluorescent images analysis. Scale bars: 200 µm. **d**, Prestoblue assay was performed to quantified the relative cell viability of fibroblasts that were grown in tissue culture dish (control), cultured on magnetic fiber, or treated with 20% DMSO for 24 hours.

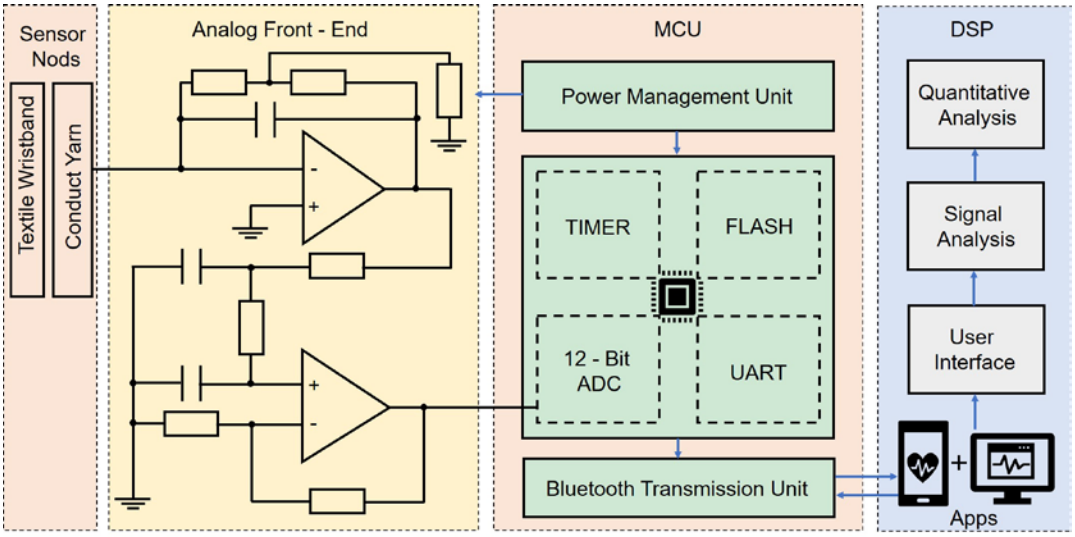

Supplementary Figure 23. System-level block diagram of the wireless wearable CMS.

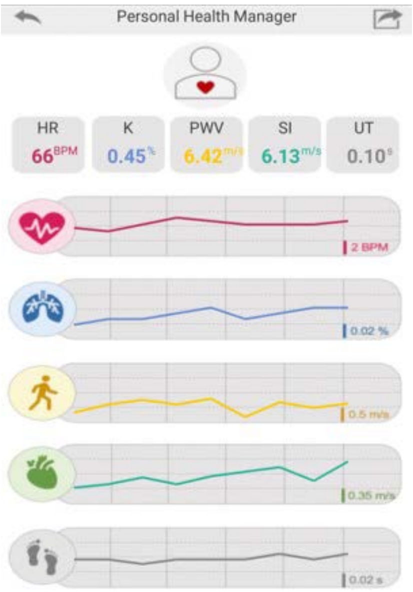

Supplementary Figure 24. Screenshot of the cellphone App showing a health table as a user interface.

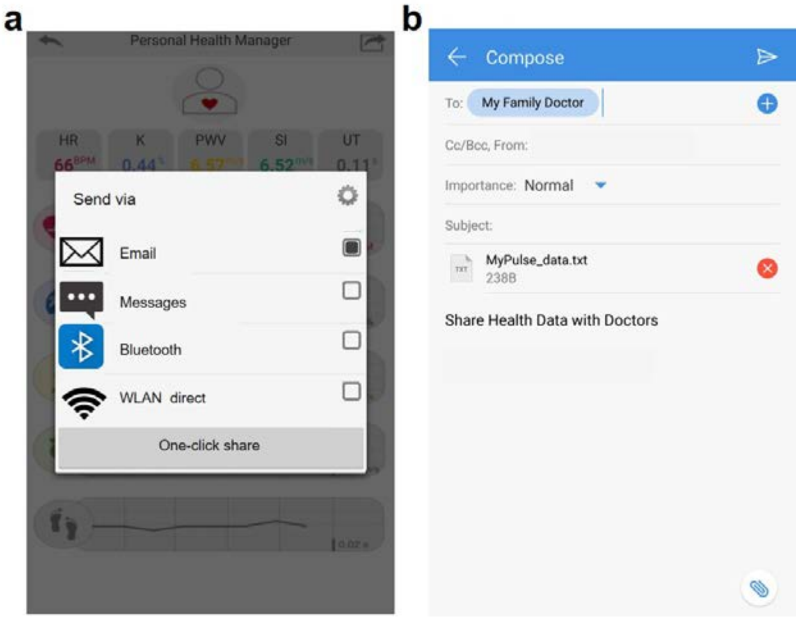

Supplementary Figure 25. Schematics of the cellphone App showing the sharing options with the physicians over a distance. a, Screenshot of the cellphone App showing the sharing options. **b**, Screenshot of the cellphone App showing the health data is sent to the physicians by email.

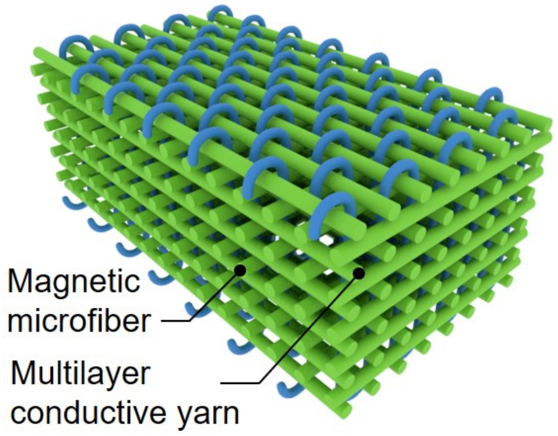

Supplementary Figure 26. Schematics of the 3D MEG textiles. The Schematics consists of multiple-layer weaved magnetic fibers and conductive yarns stacking together.

Supplementary Note 1. Detailed comparison of the magnetorheological elastomers.

The concept of magnetorheological elastomers was first proposed in 1983 when Rigbi and Jilken studied an elastomer filled with soft ferrite[1]. In the following years, research focus of magnetorheological elastomers has been placed on their magnetostriction property of changing shapes or dimensions under external magnetic field[2,3]. As a result, their applications have been mainly limited to adaptive vibration isolators and controllers for civil and mechanical engineering. In recent years, their tunable mechanical properties, such as stiffness and shear modulus under an applied magnetic field, have received great attention[4]. Through manipulating magnetic domains of hard magnetorheological elastomers in an untethered manner, magnetic soft robots with complex shape-transformability have been widely reported[5,6].

The inverse effect of magnetostriction, however, is previously ignored although it has great potential for soft-matter electronics. To our best knowledge, the mechanically induced magnetic properties change has been rarely reported in previous research. In this article, we observed the magnetoelastic effect in soft fibers with a higher magnetomechanical coupling factor than bulk metal alloys such as $Tb_xDy_{1-x}Fe_2$ (Terfenol-D)[7] and Ga_xFe_{1-x} (Galfenol)[8,9]. It is different from the traditional magnetoelastic effect in metal alloys since this effect in soft systems results from the changed particle arrangement in soft systems. Moreover, the magnetoelastic effect in our soft fibers is also distinct from pseudo-magnetoelastic effect in the iron-silicone-rubber system[10] for three reasons.

Firstly, the magnetoelastic effect in iron-silicone-rubber system requires a magnetic field of around 0.2-0.3 T, which equals 2000 to 3000 Oe. Such a static magnetic field is even larger than that typically required by the conventional rigid counterpart and therefore needs to be supplied by an electromagnet, which hinders its possibility of practical applications especially in the field of wearable and implantable bioelectronics. It is also clearly shown that without an external magnetic field, the iron-silicone-rubber system did not exhibit any magnetoelastic effect at all[10]. Therefore, instead of a true magnetoelastic effect, the studied effect in the iron-silicone-rubber system is more appropriate to be called as pseudo-magnetoelastic effect. Furthermore, the use of electromagnet will bound and prevent the leakage of the magnetic flux, which inevitably overestimate the performance of the iron-silicone-rubber system.

Secondly, the pseudo-magnetoelastic effect is studied by applying shear strain using a steel blade whereas the magnetoelastic effect is studied by applying uniaxial stress (strain), which is much more common in human biomechanical motions than shear strain. Therefore, our studies are more universal for bioelectronics applications.

Third and most importantly, the working mechanisms of pseudo-magnetoelastic effect and magnetoelastic effect are different. For pseudo-magnetoelastic effect, the magnetic flux density change is caused by the change of apparent permeability (susceptibility) under an external magnetic field. As a result, there is an optimal magnetic field around 0.2 T to achieve the best output performance. When the applied magnetic field is 0 T, there is no observable magnetoelastic effect. When the applied magnetic field is high enough (~0.7 T) to saturate the iron particle, the magnetoelastic effect diminishes because the permeability of the iron-silicone-silicone system will not change in such a situation. On the contrary, the magnetoelastic effect in our system relies on the arrangement change of readily magnetized nanomagnets. It does not require an external magnetic field and in principle will not be affected by the magnetization saturation of microparticles. As a result, the theoretical model of our system is significantly different from the one used in the iron-silicone rubber system. The theory used in the iron-silicone-rubber system cannot explain the magnetic flux decrease of our system under uniaxial stress, since in this case the particle interaction should increase with decreased particle inter-distance. By adopting the wavy chain microstructure and demagnetizing factor, our theoretical model was able to explain the observed negative magnetoelastic effect.

Supplementary Note 2. Theoretical explanation of the wavy chain analytical model.

The magnetoelastic effect in soft magnetic fibers can be described using wavy chain model with dipole-dipole interaction and demagnetizing factor. For the dipole-dipole interaction, we assume that under impulse magnetization, the nanomagnets, which can be approximately regarded as single magnetic dipoles[11], align in a zig-zag wavy chain structure as illustrated in Fig. 2i. We further assume that dipole-dipole interaction exist only inside the wavy chain according to previous literature[12]. To simplify the calculation, we consider that each magnetic dipole has a vertical magnetization *M* which is reasonable owing to the system symmetry. For the demagnetizing factor, we approximate the wavy chain as a square rod with aspect ratio of a. Based on the above assumption, the vertical magnetic field of a micromagnet on the surface of the fiber can be approximately expressed as,

$$H_{\perp} \approx \frac{1}{\chi}M - \frac{k}{2a\lambda^{1.5}+1}M + \frac{r^3M}{3\lambda^3h^3}(0.3006 - f\left(\frac{l}{h\lambda^{1.5}}\right)) \quad (1)$$

$$f\left(\frac{l}{h\lambda^{1.5}}\right) = \sum_{n=1}^{\infty} \left((\frac{l}{h\lambda^{1.5}})^2 - 2(2n-1)^2\right)(\left(\frac{l}{h\lambda^{1.5}}\right)^2 + (2n-1)^2)^{-5/2} \quad (2)$$

where λ is the principal stretch in the compress direction and *r* is the radius of the nanomagnets. *h* and *l* denote the vertical and horizontal distances between two adjacent magnetic dipoles in the wavy chain, respectively. 0.3006-*f(x)* is the dipole alignment factor describing the contribution of all other magnetic dipoles to vertical magnetic field of the single dipole on the surface of the fiber in the wavy chain. $\frac{1}{2a+1}$ represents the demagnetizing factor of the wavy chain structure. $\frac{1}{\chi}$ is a constant averaging the contribution of remnant magnetization to the overall magnetic field. *k* represents a constant characterizing the influence of nonideality, neighboring chain-chain interaction, and macroscopic shape effect to the demagnetizing factor under compressive deformation. Then the variation of vertical magnetic field due to elastic deformation of the soft magnetic system can be expressed as below,

$$H_{1\perp}/H_{0\perp} = \frac{\frac{1}{\chi} - \frac{k}{2a\lambda^{1.5}+1} + \frac{r^3}{3\lambda^3h^3}(0.3006 - f\left(\frac{l}{h\lambda^{1.5}}\right))}{\frac{1}{\chi} - \frac{k}{(2a+1)} + \frac{r^3}{3h^3}(0.3006 - f\left(\frac{l}{h}\right))} \quad (3)$$

With estimated values *of* χ =14.99, a =105, *r* = 2.5 μm, *h* = 13.5 μm, *l* = 14.85 μm and *G* = 630 kPa for the soft magnetic fiber, and the compressive stress *s* through an incompressible Neo Hookean model below,

$$s = G(\lambda - 1/\lambda^2) \tag{4}$$

When k equals 3.8, the wavy chain model accurately captures its magnetic field variation in response to the compressive stress changing from 0 to 450 kPa, which is well consistent with the experimental observation in Fig. 1g.

The introduction of k into the equation 1 is based on three reasons: It is worth noting that the derived $H_{1\perp}/H_{0\perp}$ only approximately represent the ideal case in which the edging effect and the shape of magnetic fiber were not considered. 1. The ideal rectangular rod with uniform magnetization only roughly approximate the wavy chain structure. 2. The influence of neighboring wavy chains to the demagnetizing factor of a single wavy chain cannot be simply ignored. 3. The macroscopic shape effect determines that the demagnetizing factor will not change only based on the shape of the wavy chain structure. This macroscopic effect needs to be unified inside the demagnetizing factor. It should also be mentioned that the theoretical consideration includes the contribution of remnant magnetization and the correcting parameter k is therefore placed in a different position of the equation. Both of the adjustments make the theoretical consideration more physically reasonable. Since the theory is based on a lot of assumptions and simplifications, it only roughly approximates the experimental results. More sophisticated theory should be developed in the future to better address the magnetoelastic effect in soft magnetic systems.

Supplementary Note 3. Calculation of energy conversion efficiency.

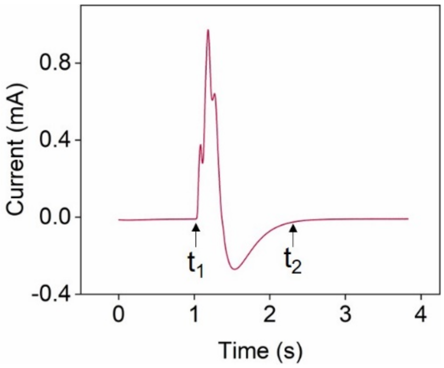

Supplementary Figure 27. The current output of the textile MEG when it was connected with an external resistance.

As an important figure of merit, the energy conversion efficiency of the textile MEG was calculated, the energy conversion efficiency is calculated by the ratio between the electric energy delivered to the external load and the mechanical energy exerted on the textile MEG. Supplementary Fig. 27 demonstrates the current output of the textile MEG when it was connected with an external resistance. The electric energy delivered by the textile MEG is equal to the Joule heat of the external resistance, which is presented below.

$$E_{electric} = R \cdot \int_{t_1}^{t_2} I^2 \cdot d_t = 1.24 \times 10^{-2}\ \text{mJ} \quad (1)$$

Where R is the external load resistance, I is the current. t_1 and t_2 is the time shown in Supplementary Fig. 27. The energy exerted by the movable substrate is calculated by the following equations.

$$E_{mechanial} = \frac{1}{2} \cdot m \cdot v^2 = 0.196\ \text{mJ} \quad (2)$$

Where m is the mass of the movable substrate (m = 2 g), and v (v = 0.14 m s^{-1}) is the velocity of the substrate when the contact between the movable substrate and textile MEG begins. Thus, the energy efficiency is calculated as

$$\eta = \frac{E_{electric}}{E_{mechanial}} \times 100\% = 6.30\ \% \quad (3)$$

Supplementary Note 4. Cardiovascular parameters analysis.

The circulation of blood will transmit oxygen and nutrient within body. Heart rate is an essential parameter during this process. Heart rate can be detected when heart pumps the blood, which generates pulse waves. Using textile MEG to percept tiny pressure fluctuations of the blood vessel, high-quality electrical signals can be obtained. After calculating the average heartbeat in one minute, we can obtain the heart rate. A normal heart rate is usually between 45-90 beats per minute.

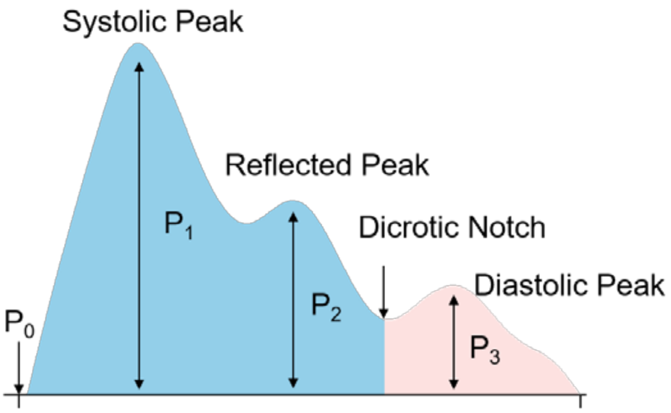

Supplementary Figure 28. A typical pulse wave profile in one cardiac cycle obtaining from our textile wristband.

As shown in Supplementary Figure 28, in one single pulse, there are three peaks, i.e., systolic peak (P_1), reflected peak (P_2), and diastolic peak (P_3). In this work, we characterize K values, pulse wave velocity (PWV), stiff index (SI) and upstroke time (UT) by analyzing the parameters in the pulse wave profile (Supplementary Figure 29). K values indicates the mean arterial blood pressure, which can be calculated by the following two equations,

$$P_m = \frac{1}{t}\int_0^t P(t)dt \quad (1)$$

$$K = \frac{P_m - P_0}{P_1 - P_0} \quad (2)$$

where P_m is the integration of the peak. P_0 and P_1 can be acquired from the pulse wave profile shown in Supplementary Figure 28. For healthy adults, the K value is normally less than 0.45.

PWV reflects the elasticity and compliance of the artery, which can reflect the degree of arterial stiffness. Larger PWV value indicates possibility of severe stiffness. The PWV values are calculated by using the following equation,

$$PWV = 0.8 \times \frac{2\Delta L}{RWTT} \quad (3)$$

ΔL is the distance from jugulum to pubic symphysis, whereas $RWTT$ is the time between systolic peak and reflected peak. The normal distribution of the PWV values is between 3.8 to 9.2 m s^{-1}, and the mean PWV value is 6.5 m s^{-1}.

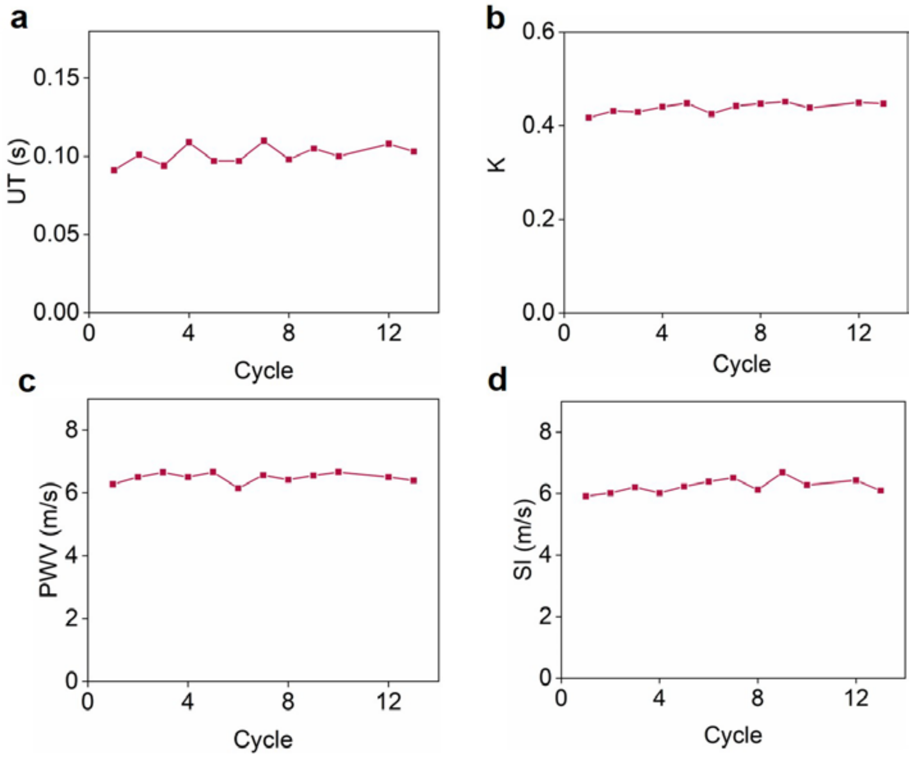

Supplementary Figure 29. **Measured pulse wave profiles obtained from textile wristband. a,** Characteristic UT obtained from pulse wave profiles. **b,** Characteristic K obtained from pulse wave profiles. **c,** Characteristic PWV obtained from pulse wave profiles. **d,** Characteristic SI obtained from pulse wave profiles.

The SI is sensitive to the artery stiffness and was calculated based on the following equation,

$$SI = \frac{H}{PPT} \quad (4)$$

H is the height of the subject, whereas PPT is the time between P_1 and P_3. In general, the SI value is less than 10 m s^{-1}. UT represents the ascending time of the systolic stage, which can be obtained by calculating the time interval between the P_0 and P_1.

Supplementary Table 1. Comparison of magnetoelastic effect in metal alloys and the textile MEGs

Material category		Young's modulus	Required external magnetic field (Oe)	Magneto-mechanical coupling factor (T/Pa)	Applied pressure range (kPa)
Fe-Co alloy		200 GPa[a]	25[c]	1.25×10^{-8} [a]	5000-70000
Co		209 GPa[a]	10[c]	2.45×10^{-8} [a]	-
Terfenol-D		19 GPa[a]	950[c]	3.26×10^{-8} [a]	11000-41000
Galfenol		54 Gpa[a]	50[c]	2.13×10^{-8} [a]	10000-50000
MEG	NdFeB[e]	629.76 kPa[b]	0	1.05×10^{-7} [d]	0-450
	Fe_3O_4[e]	303.12 kPa[b]	0	2.96×10^{-9}	0-450
	$SrFe_{12}O_{19}$[e]	834.48 kPa[b]	0	8.37×10^{-9}	0-450

[a]Estimated value. [b]Soft magnetic fiber with 83 wt% concentration. [c]Calculated based half of the saturation. [d]Calculated based maximum value on side surface. [e]Made by 83 wt% of magnetic concentrations.

Supplementary Table 2. Price of the magnetic fiber and conductive yarn.

**Material category**	**Price**
Magnetic fiber	\$0.1/m
Conductive yarn	\$0.023/m

Supplementary Table 3. Water vapor transmission rate of different textile samples.

**Textile category**	**Water vapor transmission rate**
Cotton	0.015 g cm^{-2} h^{-1}
Polyamide film	0.014 g cm^{-2} h^{-1}
Magnetic film	0.003 g cm^{-2} h^{-1}
Our device	0.018 g cm^{-2} h^{-1}

Supplementary References

1. Rigbi, Z. & Jilkén, L. The response of an elastomer filled with soft ferrite to mechanical and magnetic influences. *J. Magn. Magn. Mater.* **37**, 267-276 (1983).
2. Guan, X., Dong, X. & Ou, J. Magnetostrictive effect of magnetorheological elastomer. *J. Magn. Magn. Mater.* **320**, 158-163 (2008).
3. Deng, H.X. & Gong, X.L. Adaptive tuned vibration absorber based on magnetorheological elastomer. *J. Intell. Mater. Syst. Struct.* **18**, 1205-1210 (2016).
4. Zhao, R., Kim, Y., Chester, S.A., Sharma, P. & Zhao, X. Mechanics of hard-magnetic soft materials. *J. Mech. Phys. Solids* **124**, 244-263 (2019).
5. Kim, Y., Yuk, H., Zhao, R., Chester, S.A. & Zhao, X. Printing ferromagnetic domains for untethered fast-transforming soft materials. *Nature* **558**, 274-279 (2018).
6. Kim, Y., Parada, G.A., Liu, S. & Zhao, X. Ferromagnetic soft continuum robots. *Sci. Robot.* **4**, eaax7329 (2019).
7. Su, Q., Morillo, J., Wen, Y. & Wuttig, M. Young's modulus of amorphous Terfenol-D thin films. *J. Appl. Phys.* **80**, 3604-3606 (1996).
8. Deng, Z. & Dapino, M.J. Review of magnetostrictive materials for structural vibration control. *Smart Mater. Struct.* **27**, 113001 (2018).
9. Datta, S., Atulasimha, J., Mudivarthi, C. & Flatau, A.B. Stress and magnetic field-dependent Young's modulus in single crystal iron-gallium alloys. *J. Magn. Magn. Mater.* **322**, 2135-2144 (2010).
10. Diguet, G., Sebald, G., Nakano, M., Lallart, M. & Cavaillé, J.-Y. Magnetic particle chains embedded in elastic polymer matrix under pure transverse shear and energy conversion. *J. Magn. Magn. Mater.* **481**, 39-49 (2019).
11. Borbáth, T., Günther, S., Yu Borin, D., Gundermann, T. & Odenbach, S. XμCT analysis of magnetic field-induced phase transitions in magnetorheological elastomers. *Smart Mater. Struct.* **21**, 105018 (2012).
12. Han, Y., Hong, W. & Faidley, L.E. Field-stiffening effect of magneto-rheological elastomers. *Int. J. Solids Struct.* **50**, 2281-2288 (2013).
