# Supplementary material for: Soft fibers with magnetoelasticity for wearable electronics
Source: Nat Commun. 2021 Nov 19;12:6755. doi: 10.1038/s41467-021-27066-1 (PMC8604991; doi:10.1038/s41467-021-27066-1)
Supplement: Supplementary file 2 — Description of Additional Supplementary Files [file 41467_2021_27066_MOESM2_ESM.pdf]

Description of Additional Supplementary Files

File Name: Supplementary Movie 1
Description: 3D tomography of the soft magnetic microfiber

File Name: Supplementary Movie 2
Description: Artificial perspiration droplets absorbed by MEG textile

File Name: Supplementary Movie 3
Description: Wirelessly measuring human pulse wave in a sweaty state

File Name: Supplementary Movie 4
Description: Textile fabrication by a weaving loom
